# Simulation and forecasting models of COVID-19 taking into account spatio-temporal dynamic characteristics: A review

**DOI:** 10.3389/fpubh.2022.1033432

**Published:** 2022-10-18

**Authors:** Peipei Wang, Xinqi Zheng, Haiyan Liu

**Affiliations:** ^1^School of Information Engineering, China University of Geosciences, Beijing, China; ^2^Technology Innovation Center for Territory Spatial Big-Data, MNR of China, Beijing, China; ^3^School of Economic and Management, China University of Geosciences, Beijing, China

**Keywords:** COVID-19, spatio-temporal modeling, compartment model, metapopulation model, cellular automaton, agent based modeling

## Abstract

The COVID-19 epidemic has caused more than 6.4 million deaths to date and has become a hot topic of interest in different disciplines. According to bibliometric analysis, more than 340,000 articles have been published on the COVID-19 epidemic from the beginning of the epidemic until recently. Modeling infectious diseases can provide critical planning and analytical tools for outbreak control and public health research, especially from a spatio-temporal perspective. However, there has not been a comprehensive review of the developing process of spatio-temporal dynamic models. Therefore, the aim of this study is to provide a comprehensive review of these spatio-temporal dynamic models for dealing with COVID-19, focusing on the different model scales. We first summarized several data used in the spatio-temporal modeling of the COVID-19, and then, through literature review and summary, we found that the existing COVID-19 spatio-temporal models can be divided into two categories: macro-dynamic models and micro-dynamic models. Typical representatives of these two types of models are compartmental and metapopulation models, cellular automata (CA), and agent-based models (ABM). Our results show that the modeling results are not accurate enough due to the unavailability of the fine-grained dataset of COVID-19. Furthermore, although many models have been developed, many of them focus on short-term prediction of disease outbreaks and lack medium- and long-term predictions. Therefore, future research needs to integrate macroscopic and microscopic models to build adaptive spatio-temporal dynamic simulation models for the medium and long term (from months to years) and to make sound inferences and recommendations about epidemic development in the context of medical discoveries, which will be the next phase of new challenges and trends to be addressed. In addition, there is still a gap in research on collecting fine-grained spatial-temporal big data based on cloud platforms and crowdsourcing technologies to establishing world model to battle the epidemic.

## Introduction

The prevention and control of infectious diseases is a major topic in the field of public health all over the world. Research on the mechanism and development of infectious diseases in space is an important task for infectious diseases researchers (1). Human beings live in an increasingly interconnected world, and thanks to the convenient flights, both humans and pathogens can be brought to any city in any country (2, 3). During the spread of infectious diseases, the spatial movement of individuals will cause changes in the number of infections in different regions, which will lead to the spread of infectious diseases in large area (4, 5). To describing the spreading laws and process trends of infectious diseases in space, it is necessary to use the theory of spatial dynamics modeling to reflect the spatio-temporal characteristics and internal information of infectious diseases, and to provide theoretical guidance and policy evaluation for the prevention and control of infectious diseases (6).

The dynamics of infectious diseases is an important method to theoretically and quantitatively study the epidemic law of infectious diseases (7, 8). It is based on the characteristics of population growth, the law of disease occurrence and transmission through the population and other relevant factors to establish a mathematical model that can reflect the dynamic characteristics of infectious diseases. The dynamics characteristic model based on qualitative and quantitative analysis and numerical simulation can reveal its epidemic law, predicting its changing trend, and analyze its epidemic reasons and key factors. Ultimately, dynamic models could seek the optimal prevention and control strategy, and provide theoretical and quantitative basis for people to make prevention and control decisions (9). Compared with traditional biostatistics methods, the dynamic methods can better reflect the epidemic law in terms of the transmission mechanism of the disease, so that people can understand some global states in the epidemic process (10, 11). The combination and complementation of infectious disease dynamics, biostatistics, computer simulation and other methods have enabled people to have a more in-depth and comprehensive understanding of the laws of epidemic disease, making the established theories and prevention strategies more reliable and practical (12, 13).

Neither traditional Ordinary Differential Equations (ODEs), time-lag ODEs or statistical methods nor clinical case studies consider the spatial diffusion of individuals (14). In the face of highly contagious and highly pathogenic infectious diseases such as COVID-19, from the Delta variant to the Omicron variant, each mutation of COVID-19 will trigger a new round of prevention and control tensions (15). Behind the vigorous prevention and control, it has caused immeasurable losses to the normal life of the people and social and economic development (16, 17). For COVID-19, the spatial movement of individual groups is a key factor leading to the rapid spread of infectious diseases (4, 18). Therefore, the study of spatial diffusion models can better reflect the spread dynamics of COVID-19 in time and space. It is worth mentioning that the impact of medical indicators on the modeling of the spatio-temporal dynamics of COVID-19 spread varies at different stages of its development. In the early stage of the epidemic, the speed and scale of the spread of the epidemic are largely depended on the ability of testing and reporting and hospital treatment capacity (19). In the mid-term, medical parameters (including incubation period (20), reproductive number (21), mortality rate (22), etc.) can be promoted with the help of the abundant epidemic statistical data of the public health departments to provide a basis for parameter selection and optimization of the spatio-temporal dynamics evolution of infectious diseases. Late-stage data is abundant, combined with medical interventions (vaccination, virus detection, etc.), which can provide the possibility for precise prediction of spatio-temporal dynamics modeling (23).

As COVID-19 continues to evolve, the number of models studying its spatio-temporal changes has exploded. A study on geospatial technologies (24) looked at COVID-19 from an interdisciplinary perspective and reviewed relevant academic results published up to the end of May 2020, while a research review (25) reviewed the literature published up to the end of September 2020, points to geospatial analysis in COVID-19 research and highlights current trends and research gaps. In a paper published in 2020, we extend the previous forecast period and accuracy with the help of Logistic and Artificial Intelligence (AI) (26). The publication of this paper attracted the attention of many peers and became a hot and highly cited paper on the Web of Science (WOS). At the same time, many deep learning methods have been used for COVID-19 prediction and spatio-temporal feature processing. Researchers investigated the performance of the Random Forest (RF) machine learning algorithm in estimating the near future case numbers for 190 countries in the world (27). Jin et al. (28) study the spatial-temporal characteristics of the epidemic development at the provincial-level in mainland China and the civic-level in Hubei Province. And Karadayi et al. (29) proposed a hybrid deep learning framework to solve the unsupervised anomaly detection problem in multivariate spatio-temporal data. However, based on our research and tracking of international research progress, we found that in the summary of the COVID-19 evolution prediction model, the spatio-temporal dynamic model with large development space and application prospects has not been well summarized. For humanity to better respond to COVID-19, it is necessary to review the progress that has been made and recommend further research to enable more people to work together in public relations to help deliver a targeted response to COVID-19 as quickly as possible measures.

This study will further integrate the spatial model of COVID-19 based on previous research, focusing on the analysis of the spatio-temporal model related to COVID-19, so as to facilitate readers to sort out. This paper classifies and summarizes the COVID-19 spatio-temporal dynamics model. According to the modeling basis and modeling principles, macro-dynamic models and micro-dynamic models (Table 1) are summarized. Macro-dynamic models usually include compartmental models and metapopulation models. The population is divided into several compartments, which represent different diseases states of the population, and mathematical methods are used to establish its dynamic equations to simulate and study the process of transmission dynamics. The latter are mainly based on Cellular Automaton (CA) and Agent Based Modeling (ABM). An individual is thought of in a population as a cellular unit or agent consisting of a limited set of rules of state and behavior. By defining the corresponding behavior of the individual to the etiology, the mobile behavior of individual in the space and the interaction between the individual behavior rules, the behavior of complex systems such as infectious diseases that evolve from the cause, host, and environment can be simulated. In addition, the collection and organization of spatio-temporal big data is the basis for establishing spatio-temporal dynamics models of infectious diseases, and is also the cornerstones of studying spatio-temporal dynamics models. Here, we review different efforts to model the spread of COVID-19, which should stimulate new thinking and facilitate the integration of geographic information and computer technology into infectious diseases modeling and control. Our main research objectives will to be:

1. To review the application of spatio-temporal big data in spatial modeling of COVID-19.
2. Classify the infectious disease transmission model into macro and micro models, analyze the advantages and disadvantages of various modeling methods, and highlight current spatio-temporal modeling methods.
3. Analyze the role of spatio-temporal modeling in COVID-19 research by comparing the results of model modeling at different scales.
4. Highlight current research trends and gaps of COVID-19 spatio-temporal modeling.

Our research contributions are as follows:

1. Due to previous experience, more studies are carried out with the help of existing models, but less new models are developed. Because people's judgment of the epidemic is that “short and smooth” approach is needed, the development of new models is blocked. At present, all relevant models proposed by researchers can be concluded as macro-dynamics model and micro-dynamics model.
2. Future research direction of spatio-temporal dynamics model of infectious diseases should be to introduce multi-level and multi-scale integrated epidemic spatio-temporal dynamics model with big data and intelligent computing. Moreover, these models are adaptive, that is, they can quickly simulate and predict adaptability according to the situation of different countries and provide quick reference for management and control decisions.
3. The integration of macro and micro models and adaptive medium and long term (from months to year) spatio-temporal dynamic simulation decision support model combined with medical conclusions will be new challenges and trends to be addressed in the next stage.

**Table 1:** References to macro- and micro-dynamic simulation models of COVID-19 are grouped according to research focus, main research findings, models, method description, data, and geographic scope.

**References**	**Study focus**	**Main findings**	**Models**	**Method description**	**Data**	**Geographic extent**
**Macro-dynamic model**						
He et al. (30)	Epidemic prediction of COVID-19	The parameters of the proposed SEIR model are different for different scenarios.	SEIR; PSO	A SEIR epidemic model was built according to some general control strategies. And the PSO algorithm is applied to estimate the parameters of the system.	COVID-19 epidemiological data	Hubei, China
Team (31)	Assessing scenarios of social distancing mandates and levels of mask use	Achieving universal mask use (95% mask use in public) could be sufficient to ameliorate the worst effects of epidemic resurgences in many states.	SEIR model	Modeling deaths and cases across three boundary scenarios of social distancing mandates in US states using an SEIR framework.	COVID-19 case and mortality data from 1 February 2020 to 21 September 2020	in the United States at the state level
Cui et al. (32)	Epidemic prediction of COVID-19	The quarantine magnitudes in the susceptible individuals play larger roles on the disease control than the impacts of the quarantines of the exposed individuals and infectious individuals. The early quarantine strategy is significantly important for the disease controlling.	SEIRQ model	The whole population at time t was divided into seven compartments which include the susceptible *S*(*t*), exposed *E*(*t*), infectious *I*(*t*), removed *R*(*t*), quarantined susceptible *Sq*(*t*), quarantined exposed *Eq*(*t*) and quarantined infectious individuals *Iq*(*t*).	COVID-19 epidemiological data	Wuhan and mainland China
Liu et al. (33)	Epidemic prediction of COVID-19	China's national policies had effectively slowed down the spread of the epidemic.	SEIRD model, LSTM and GWR	After proposing the improved model of SEIRD by adding a decay factor to the infection rate β, LSTM and GWR models are directly applied in this paper.	COVID-19 epidemiological data; The daily number of outbound from Wuhan city and relevant migration indices from January to March; The demographic data and medical resources data	China
Godio et al. (34)	The application of a stochastic approach in fitting the model parameters using a Particle Swarm Optimization (PSO)	Discussed the effectiveness of policies taken by different regions and countries.	SEIR model; PSO algorithm	A generalized SEIR model was adopted and the parameters were fitted in a least-square sense with a deterministic approach, and then with a stochastic approach, using a PSO algorithm.	COVID-19 epidemiological data	Italy and its Lombardy, Piedmont, and Veneto regions
Yang et al. (35)	Predicting the COVID-19 epidemic peaks and sizes	The epidemic of China should peak by late February, showing gradual decline by end of April in 2020.	SEIR model; LSTM	Population migration data and most updated COVID-19 epidemiological data are integrated into the SEIR model to derive the epidemic curve. And LSTM model is trained on the 2003 SARS data, to predict the epidemic of COVID-19.	COVID-19 epidemiological data; Migration index; 2003 SARS epidemic data	China
Wang et al. (36)	Super-spreading events	Super-spreading events played an important role in the early stage of the COVID-19 break.	Phylogenetic analysis with Bayesian inference; SEIR model	Analyzing 208 publicly available SARS-CoV-2 genome sequences collected during the early outbreak phase. Then, combining phylogenetic analysis with Bayesian inference under an SEIR model to trace person-to-person transmission.	208 publicly available SARS-CoV-2 genome sequences	Wuhan, China
Chen et al. (37)	Simulating the phase-based transmissibility of covid-19	The transmissibility of SARS-CoV-2 was higher than the Middle East respiratory syndrome in the Middle East countries, similar to severe acute respiratory syndrome, but lower than MERS in the Republic of Korea.	Bats-Hosts-Reservoir-People transmission network model	Simplified the Bats-Hosts-Reservoir-People model as Reservoir-People (RP) transmission network model. The next generation matrix approach was adopted to calculate the basic reproduction number (R0) from the RP model to assess the transmissibility of the SARS-CoV-2.	COVID-19 epidemiological data	China
**Micro-dynamic model**						
Balcan et al. (38)	Simulate the spread of epidemics at the worldwide scale	Build discrete stochastic epidemic model.	GLEaM	GLEaM integrates three different data layers: population layer, transportation mobility layer and disease model. Based on a metapopulation approach in which the world is divided into geographical regions defining a subpopulation network where connections among subpopulations represent the individual fluxes due to the transportation and mobility infrastructure.	Epidemiological data; sociodemographic and population mobility data	Worldwide scale
Chinazzi et al. (39)	Project the impact of travel limitations on the national and international spread of the epidemic	The travel quarantine of Wuhan delayed the overall epidemic progression by only 3 to 5 days in mainland China but had a more marked effect on the international scale, where case importations were reduced by nearly 80% until mid-February.	GLEaM	By introducing GLEaM, the world is defined in geographical census areas connected in a network of interactions by human travel fluxes corresponding to transportation infrastructures and mobility patterns.	Airline data, epidemiological data and sociodemographic	Worldwide scale
Ghosh and Bhattacharya (40)	Identify the major factors of this infection spreading dynamics	A probabilistic cellular automata based method was proposed to model the infection dynamics for a significant number of different countries.	CA; GA; SEIR	The parameters of CA model of cellular automata were optimized by sequential evolutionary genetic algorithm, and the epidemic development curve was simulated.	COVID-19 epidemiological data and population data	Worldwide scale
Monteiro et al. (41)	The actual role of immune individuals in infection spread	Immune individuals affect the spread of contagious diseases.	CA; GA	By using a genetic algorithm, the values of three parameters of CA model are determined from data of prevalence of varicella in Belgium and Italy, in a pre-vaccination period.	Data of varicella prevalence	Belgium and Italy
Monteiro et al. (42)	Dynamic modeling of COVID-19	Evaluate the impact of distinct quarantine regimes on the SARS-CoV-2 pandemic.	PCA; ODE model	From its mean-field approximation written in terms of ODE, analytical expressions for the basic reproduction number and the ratio between symptomatic and symptomatic individuals were derived. Then, those expressions be used to characterize the dynamical behavior observed in computer simulations with the PCA model.	N/A	Virtual scene
Medrek and Pastuszak (43)	Infectious disease dynamics modeling	The earlier reduction of personal contacts the faster reduction of infections number.	CA; SEIR	A simulated model of COVID-19 was developed using an improved influenza transmission model and incorporating factors related to the actual demographic and geographic profile of the simulated population.	COVID-19 epidemiological data; population density and age structures	Poland, France and Spain
Ghosh and Bhattacharya (44)	Understand the effects of these measures on the control of the epidemic in a data-driven manner	Gives us both spatial and temporal variations of the infection spread with the insight about the contributions of different infection parameters.	PCA;	A spatially explicit epidemiological model through PCA on a square lattice with SEIQR structure is defined. Instead of having three subpopulations, COVID-19 has partitioned the society in five different subpopulations.	N/A	Virtual scene
Jizhe et al. (45)	Modeling epidemic dynamics	The high intensity of population movement in the Greater Bay Area (GBA) brings a high risk of virus transmission. All kinds of control measures taken by the epidemic prevention department have a strong restraining effect on the spread of the virus in the GBA.	SEIR model	By fusing multi-source space-time dynamic city big data, build the new improvement of epidemic risk assessment model, and implement a large bay area of guangdong nine cities of multi-scale risk assessment is the spread of the virus.	COVID-19 epidemiological data; Census data; Cellular signaling data; Point of Interests (POI)	GBA
Ferretti et al. (46)	Epidemic control based on contact tracing	Explored the feasibility of protecting the population (that is, achieving transmission below the basic reproduction number) using isolation coupled with classical contact tracing by questionnaires vs. algorithmic instantaneous contact tracing assisted by a mobile phone application.	Mathematical formalism	Using key parameters of epidemic spread to estimate the contribution of different transmission routes with a renewal equation formulation, and analytically determined the speed and scale for effective identification and contact tracing required to stop the epidemic.	N/A	N/A
Keskinocak et al. (47)	Project the number of COVID19 infections	Shelter-in-place followed by voluntary quarantine substantially could reduce COVID19 infections, healthcare resource needs, and severe outcomes.	ABM	ABM captures the natural history of the disease at the individual level, by age group, as well as the infection spread *via* a contact network consisting of interactions in households, peer groups (workplaces, schools), and communities, with different rates of transmission.	COVID-19-specific parameters and data from Georgia on population interactions and demographics	USA
Hinch et al. (48)	Predict the spread of infection and assess the impact of public health measures	Present OpenABM-Covid19: an agent-based simulation of the epidemic including detailed age-stratification and realistic social networks.	ABM	Workplaces, schools, and social environments are modeled as Watts Strogatz small-world networks, and homes are modeled as independent fully connected networks. The network was parameterized such that the average number of interactions matched the age-stratified data. In these interactive networks, contact between synths has the potential to spread the virus that causes COVID-19, which can then be recalled for contact tracing and possible isolation.	COVID-19 epidemiological data; demographics data; observed hospitalization data; seroprevalence data	UK
Kerr et al. (49)	Project epidemic trends, explore intervention scenarios, and estimate resource needs	Covasim was developed to help policymakers make decisions based on the best available data.	ABM	Covasim calculates the probability that a given agent will change from one state to another at a given time step, such as from susceptible to infection, or from critically ill to dead. Once these probabilities are calculated, a pseudorandom number generator with user-specified seeds is used to determine whether a transition actually occurred during a given model run.	Data on country age distributions and household sizes as reported by the UN Population Division 2019	Africa, Asia-Pacific, Europe, and North America
Silva et al. (50)	Simulate the pandemic dynamics	In the impossibility of implementing scenarios with lockdown, which present the lowest number of deaths and highest impact on the economy, scenarios combining the use of face masks and partial isolation can be the more realistic for implementation in terms of social cooperation.	ABM; SEIR	By emulating a closed society living on a shared finite environment, composed of humans, which are organized in families, business and government, which interact with each other. COVID-ABS try to cover the main elements of the society.	Social, demographic and economical parameters	Brazil
Wei et al. (51)	Simulate the spatial spread of an epidemic	Constructed a city-based epidemic and mobility model (CEMM) to stimulate the spatio-temporal of COVID-19.	ABM	The adapted CEMM takes the city as the basic unit of analysis, assumes each city as the agent node, takes the total population of the city, the number of infected cases and other parameters as node attributes, and takes the population flow between cities as the connection between nodes to build a multi-agent network, also known as the urban network.	Tencent migration data; COVID-19 epidemiological data	China
Castro and Ford (52)	Simulating COVID-19 transmission in University Students	Presented a new geospatial agent-based simulation model to explore the transmission of COVID-19 between students living in Newcastle University accommodation and the efficiency of simulated restrictions	ABM	By using the GAMA platform, a 3D ABM of buildings, footpaths, students, other dynamic and static agents is developed. Their interactions, based on daily routines, and the implementation of a mathematical epidemiological SEIR model, allowed the simulation of generic outbreaks in the area of study.	OS Mastermap building data; OS OpenMap—Local footpath data; student data	Newcastle University
Hoertel et al. (53)	Evaluate the potential impact of intervention strategies against COVID-19	Proposed a stochastic agent-based microsimulation model of the COVID-19 epidemic in New York City	ABM	The model included 148 parameters. Parameters on individual and disease characteristics (*n* = 117) were mainly based on available data from prior studies and model calibration. Parameters related to social contacts were based on either prior studies (*n* = 9) or assumptions when no data were available (*n* = 22).	COVID-19 epidemiological data; population data.	New York City

These contributions may have been mentioned by some researchers, but have not been explicitly proposed or formally published. Through literature analysis, we systematize and display them, so that more people can further study them during or after the epidemic, and provide scientific and technological support for the next major infectious disease epidemic that may be encountered by mankind.

In the subsequent sections, this article will list the spatio-temporal big data sets collected by various researchers since the outbreak of COVID-19 firstly, and explain the data basis of spatio-temporal dynamic model modeling. From the above-mentioned model classification and induction ideas, the spatio-temporal dynamics models of COVID-19 are summarized, and finally research directions for future infectious disease dynamics modeling are proposed.

## Methodology

### Data sources

Since the outbreak of the COVID-19 at the end of 2019, large number of studies on the spread of COVID-19 based on infectious disease dynamic models have been carried out around the world. To review the scope of this paper, we use the following steps to identify relevant literature for this review. First, we searched on the WOS and Google Scholar databases with the subject items (COVID- 19 OR SARSCOV-2) AND (spatial OR temporal OR spatial analysis OR space-time OR Geospatial) AND (model OR simulate OR simulation OR evaluate OR dynamic) and (transmission or propagation or spread). The literature data retrieval and setting conditions are shown in Table 2. Further, we conducted a reference search, if any, outside the scope of the database. Considering the origin of the outbreak at the end of 2019, we limited the literature search to between 2019 and 2022. The search was first conducted on April 5, 2022 and updated on August 24, 2022. We include published or peer-reviewed journal articles based on modeling of COVID-19 spatio-temporal dynamics.

**Table 2:** Literature data retrieval condition settings such as document database, retrieval mode, retrieval terms, time span, genre and language in this study.

**Literature retrieval item**	**Literature retrieval settings**
Document database	Web of Science core collection, Google Scholar
Retrieval mode	Topic search
Retrieval terms	(COVID-19 OR SARSCOV-2) AND (spatial OR temporal OR spatial analysis OR space time) AND (model OR simulate OR simulation OR evaluate OR dynamic)
Time span	January 1, 2020 to August 24, 2022
Genre	All documented types
Language	English

**Figure 1:**
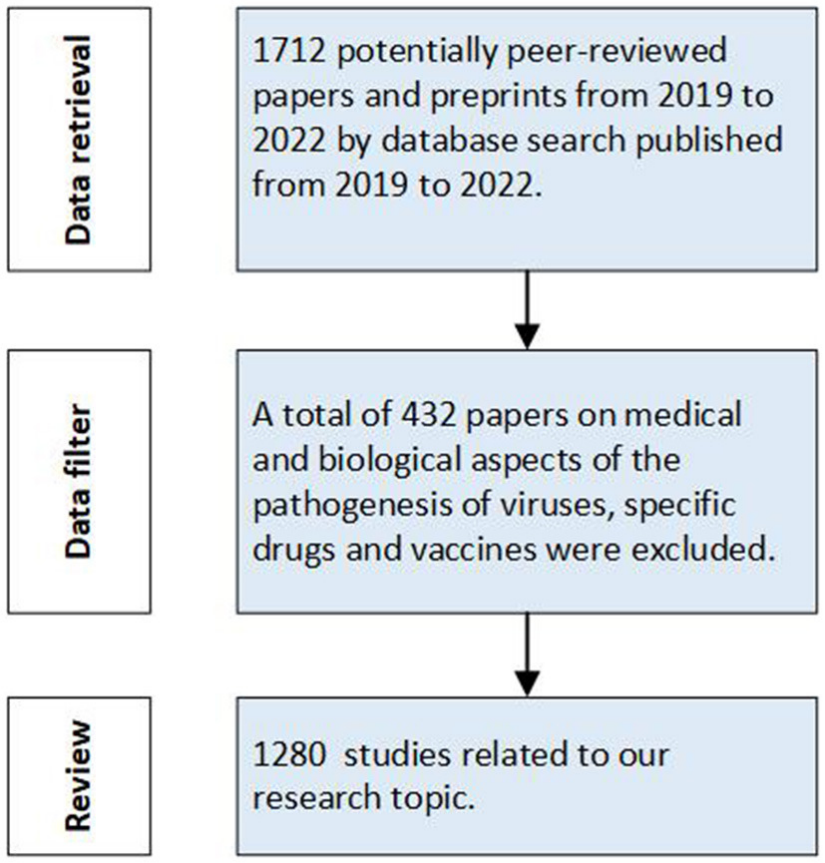
Study selection progress (Articles retrieved on August 24, 2022). Data retrieval was conducted firstly to select relative papers from 2019–2022. Then we filtered the retrieval data and reviewed the studies related to our research topic.

The progress of research selection is shown in Figure 1. According to our retrieval strategy, 1,712 papers were retrieved and categorized according to research data, scale and modeling methods, namely spatio-temporal big data, macro-dynamic modeling and micro-dynamic modeling. These studies are used for practical prevention of different stages in different countries and regions, and provide scientific weapons for human beings to resist the spread of disease.

### Eligibility criteria

Articles were considered eligible for inclusion if they extensively described techniques or reviews related to the modeling of spatio-temporal dynamics during the spread of COVID-19. We included articles in English published during the COVID-19 outbreak from 2019 to 2022, considering both qualitative and quantitative research types of manuscript. In addition, it is worth noting that papers that study some of the pathogenic mechanism of viruses, specific drugs and vaccines from a medical and biological perspectives have been removed from our study selection. Articles that were not peer-reviewed (e.g., letters, editorials, and comments) were considered ineligible for inclusion.

## Results

We mainly identified records that related to spatio-temporal big data and dynamic models of COVID-19 and made typical models of macro-dynamic and micro-dynamic models of COVID-19. Macro models usually include compartmental models and metapopulation models. Compartmental model treats the population as homogeneous mixing, while metapopulation model divides the population into several subgroups. A typical representation of micro models is an individual-based model, which treats individuals in a population as cells or agents consisting of a finite set of states and behavior rules. Primary related datasets and model summary can be analyzed below.

### Primary related datasets

The spatio-temporal dynamics modeling of infectious disease transmission mainly relies on spatio-temporal big data, which provide the data basis for the modeling, monitoring, forecasting and early warning of infectious disease transmission, and provide strong decision support for epidemic prevention and control and the resumption of work and production in more provinces and cities. Therefore, before reviewing and summarizing the modeling of spatio-temporal dynamics of infectious diseases, we first summarized and classified the spatio-temporal data of COVID-19 commonly used in these articles.

According to the characteristics and uses of the data, this paper divides the existing spatio-temporal big data of COVID-19 into three categories, epidemic statistical data, location-based data, and Non-pharmaceutical Interventions (NPIs) data. Epidemic statistical data is mainly used to support the calculation of basic indicators of the epidemic, and can be used to support the spatial and temporal statistical analysis of the epidemic and the visual analysis based on Geographic Information System (GIS), etc. The spatio-temporal location big data mainly includes Origin-to-Destination (OD) data, cellular network signaling data, and traffic network data provided by major operators such public data sets are mainly used to analyze the changes and migration of the epidemic in the spatial dimension. NPIs data mainly includes case identification, contact tracing and related measures, environmental protection measures, health care and public health capabilities, social distance, travel restrictions, etc., which can be used to measure the confrontation between society and government and the effectiveness and spatial effect of a series of NPIs for the rapid spread of COVID-19. Table 3 lists common data sources for these three types of data, as well as the corresponding indicators they provided.

**Table 3:** Grouping of COVID-19 spatio-temporal dataset sources into epidemic statistical data, spatio-temporal location data and NIPs data.

**Type**	**Data source**	**Data level**	**Items**	**URL**
Epidemic statistical data	Johns Hopkins University Center for Systems Science and Engineering (CSSE) (54)	Country	Daily confirmed, deaths and recovered cases	https://github.com/CSSEGISandData/COVID-19
	World Health Organization (WHO) (55)	Country	Cases, deaths and vaccination	https://www.who.int/emergencies/diseases/novel-coronavirus-2019
	European Center for Disease Prevention and Control (ECDC) (55, 56)	Country	Cases and deaths	https://www.ecdc.europa.eu/en/geographical-distribution-2019-ncov-cases
	Worldometer (40)	Country	Daily confirmed, deaths, recovered and tests cases	https://www.worldometers.info/coronavirus/
	US Center for Disease Prevention and Control (US CDC) (31)	County	Cases, deaths, and testing and vaccination	https://www.cdc.gov/coronavirus/2019-ncov/index.html
	COVID Tracking Project (57)	State	Cases and deaths, testing and hospitalization Data	https://covidtracking.com/data
	National Health Commission of the People's Republic of China (23, 35)	City	Confirmed, deaths and recovered cases	http://www.nhc.gov.cn/xcs/yqfkdt/gzbd_index.shtml
Spatio-temporal location data	Baidu (4, 35, 39)	City	Migration scale index	http://qianxi.baidu.com/
	SafeGraph (58)	County	The anonymous and aggregated place visit data	https://safegraph.com
	Descartes Labs (18, 58)	County	Mobility statistics (representing the distance a typical member of a given population moves in a day) at the US admin1 (state) and admin2 (county) level	https://github.com/descarteslabs/DL-COVID-19
	Google mobility reports (18, 59)	County	Movement trends over time by geography, across different categories of places such as retail and recreation, groceries and pharmacies, parks, transit stations, workplaces, and residential.	https://www.google.com/covid19/mobility?hl=en
	Apple mobility reports (18, 60)	County	Mobility trends based on location data of Apple's maps services.	https://www.apple.com/covid19/mobility
	Twitter API (18, 61)	County	The geographic location of the tweet	https://developer.twitter.com/en/docs/twitter-api/v1/tweets/sample-realtime/overview/decahose
NPIs data	Our World in Data (62)	Country	More than 200 countries with data on vaccinations, hospitalizations, risk of death, policy responses, etc.	https://ourworldindata.org/covid-cases
	OxCGRT (63)	Country	School closing, workplace closing, restriction on gatherings, stay-at-home requirements, vaccines, etc.	https://github.com/OxCGRT/covid-policy-tracker/tree/master/data
	WHO (64)	Country	Masks, schools, businesses, gatherings, domestic movements, international travel.	https://covid19.who.int/measures
	Complexity Science Hub COVID-19 Control Strategies List (CCCSL) (65)	Country	Contact tracing, environmental measures, healthcare and public health capacity, social distancing and travel restriction, etc.	https://covid19-interventions.com/

#### Epidemic statistical data

COVID-19 epidemic statistical data have been successfully applied to various epidemic prediction models in the early stage of the epidemic, and most of the early epidemic prediction models were data-driven (66, 67). Commonly used epidemiological statistics are global and country-wide, including indicators such as the number of confirmed cases, the number of death cases, the number of recovered cases, the number of tests, and the number of vaccinations, etc. By counting the number of confirmed epidemics in various regions, statistical models and mathematical models are used to predict the development trends of future epidemic (68, 69). For example, Yang et al. (35) derived the epidemic curve by integrating the latest epidemiological data and the population migration data and founded a 5-day delay would triple the size of the outbreak in mainland China. Wang et al. (54) applied the coupled mathematical model Logistic and machine learning Prephet prediction method to predict the development trend of COVID-19 in countries such as the world, Brazil, Russia, India, Peru, and Indonesia based on the statistical data collected by Johns Hopkins University. Analysis of the forecast curve shows that the response measures taken by countries in early March 2020 controlled the spread of the epidemic to a certain extent (26).

#### Location-based data

The proliferation of mobile devices has made it easier to access location-based data of COVID-19. Researchers can construct COVID-19 spatio-temporal data sets based on cellular signaling data provided by major operators, thereby mining OD data and using it to analyze the spatio-temporal spread of the epidemic (70). For example, Gao et al. (58) derived data from SafeGraph and modeled the relationship between changes in population mobility and the diagnosis rate of COVID-19. Hou et al. (5) combined human mobility and social media big data to model the spread of COVID-19 and the spread of risk. Public epidemic prevention and control services based on spatio-temporal location big data make cities more intelligent. GIS and big data technology play an important role in the rapid aggregation of multi-source big data, rapid visualization of epidemic information, spatial tracking of confirmed cases, regional transmission prediction, epidemic risk, and spatial division of prevention and control levels. It can balance the supply and demand management of material resources, eliminate social emotional panic, and provide solid spatial information support for epidemic prevention and control decision-making, measure formulation and effect evaluation (71).

In the current era of globalization, the flow of people, vehicles and logistics has shown explosive growth, making public epidemic prevention and control a global challenge. These data are typical spatio-temporal data, so the whole society urgently needs to develop public epidemic prevention and control services based on spatio-temporal location big data.

#### NPIs data

In the early stages of a pandemic, in the absence of vaccines or effective treatments, NPIs are essential. However, some of these measures have caused significant damage to social development and the national economy. NPIs such as isolation, school closures, social distancing, and wearing masks do reduce the spread of the outbreak, but the potential degree of mitigation is unclear, especially the relationship proportions of these interventions. Minimizing the impact of the epidemic and its impact on people's lives is a scientific issue that needs to be explored. Scholars and related public health institutions have collected and established a series of data sets of NPIs measures, including case identification, contact tracing and related measures, environmental measures, health care and public health capacity, resource allocation, normal life, risk communication, social distance and travel restrictions, etc.

### Model summary

**Figure 2:**
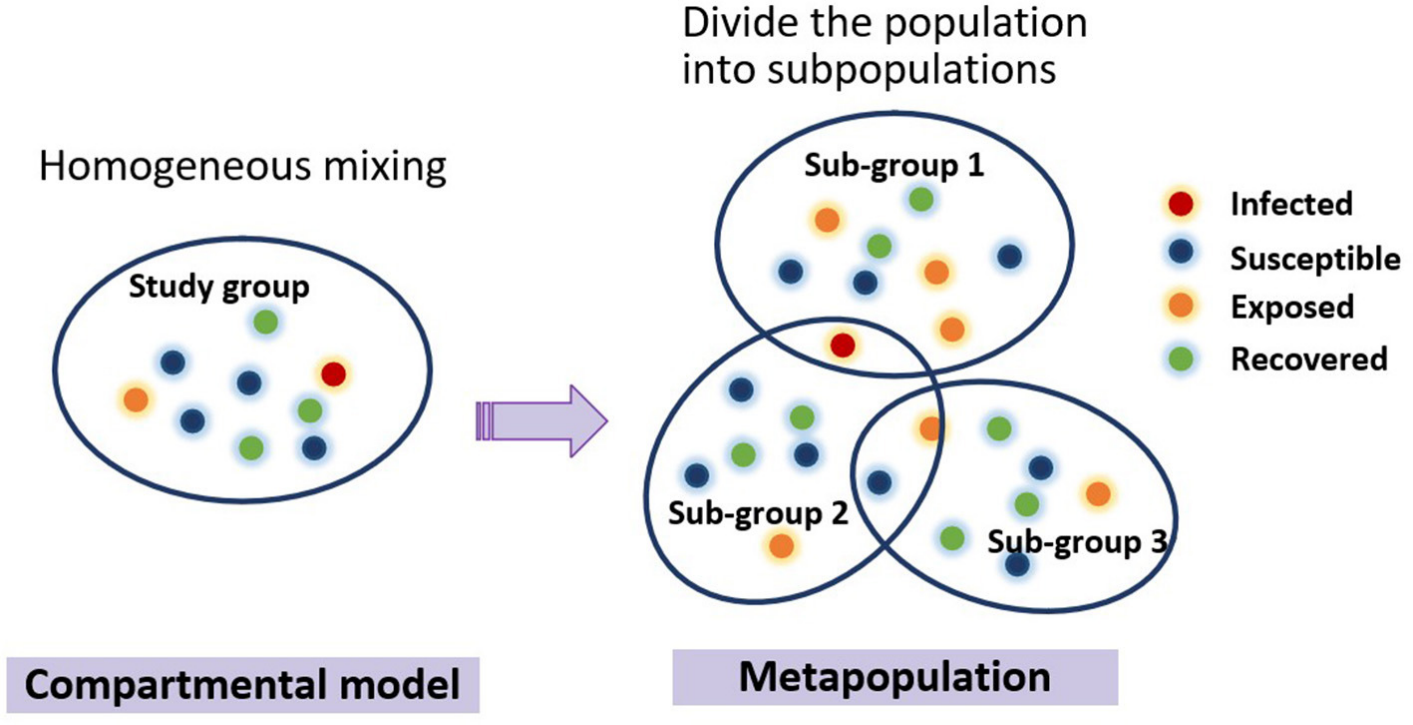
Evolution of the typical macro-dynamic models. Each circle represents an individual, and different color of the circle signifies different type of people. Compartmental model treats the population as homogeneous mixing, while metapopulation model divides the population into several subgroups. People can come and go between populations, and the compartmental model can be used to simulate the internal transmission process of infectious diseases within populations.

Through the review of the spatiotemporal dynamic propagation model in the previous section, we conclude that macro models usually include compartmental models and metapopulation models. Compartmental model treats the population as homogeneous mixing, while metapopulation model divides the population into several subgroups, as is illustrated in Figure 2. A typical representation of micro models is an individual-based model, which treats individuals in a population as cells or agents consisting of a finite set of states and behavior rules.

#### Macro-dynamic model

***Compartmental model***. In the historical development of epidemic dynamics modeling, Ross's study on malaria and Kermack and Mckendrick's study on epidemic transmission models are two important milestones (72, 73). The SIR compartmental model and its variants, such as SEIR and SIRS, are traditional compartmental models widely used to estimate the effective reproduction number and predict epidemic peak duration or inflection points, which constitute the mainstream of epidemic dynamics research (74–76). In the compartment model, different compartments are established to represent people in different disease states, and differential equations are established to study the transmission dynamics of the disease. For example, S, I and R in the SIR model represent three groups of people: susceptible, infected and recovered people, respectively.

In response to COVID-19, He et al. (30) established the SEIR model of COVID-19 based on general control strategies such as hospitals, isolation and external input, and used the model to simulate and predict the evolution of the epidemic in Hubei Province, China. Institute for Health Metrics and Evaluation (IHME), a COVID-19 modeling team at the University of Washington, constructed a SEIR model to predict the number of COVID-19 deaths in the United States from September 22, 2020 to February 28, 2021 based on data from February 1 to September 21 (31). In order to classify the disease status of the population and analyze the transmission dynamics of infection more accurately, many variants of SEIR model have been proposed by researchers, such as SEIRQ (32), SEIRQD (33), which Q and D represents quarantine and deaths, respectively. However, the virus infection rate and other parameters in those models are usually constant. It is difficult to model the spread of COVID-19 and predict the epidemic trend accurately, with 0.9448 *R*^2^ and 0.1 *MAPE* of the cumulative confirmed cases respectively. In order to solve this problem, scholars have proposed a series of methods to dynamically update and correct the parameters. Using the Particle Swarm Optimization (PSO) algorithm to solve the parameters of the SEIR model, Godio et al. (34) analyzed different scenarios of the evolution of COVID-19 in Italy, and the results showed that each region of Italy will reach the peak of the epidemic by mid-May 2020. In this method, provided models that fitted the observed data with good accuracy, although the stochastic approach has, in general, a slightly lower error. A hybrid model of SEIRD and ARIMA was also established to predict the development curve of the epidemic. The use of ARIMA model eliminates the residual of the dynamic model, improves the accuracy of the prediction of infectious diseases by the compartmental model, and prolongs the prediction period (13, 77). In addition, some scholars have combined several prevention and control measures with the SEIR model to derive epidemiological curves and evaluate the impact of various prevention and control measures on the development of the epidemic. Yang et al. integrated the migration data of Wuhan, Hubei province around January 23, 2020 into the SEIR model, and found that the epidemic in China should peak at the end of February and gradually decline at the end of April, 2020. A delay of 5 days in shutting down Wuhan could triple the size of the outbreak in mainland China (35).

**Figure 3:**
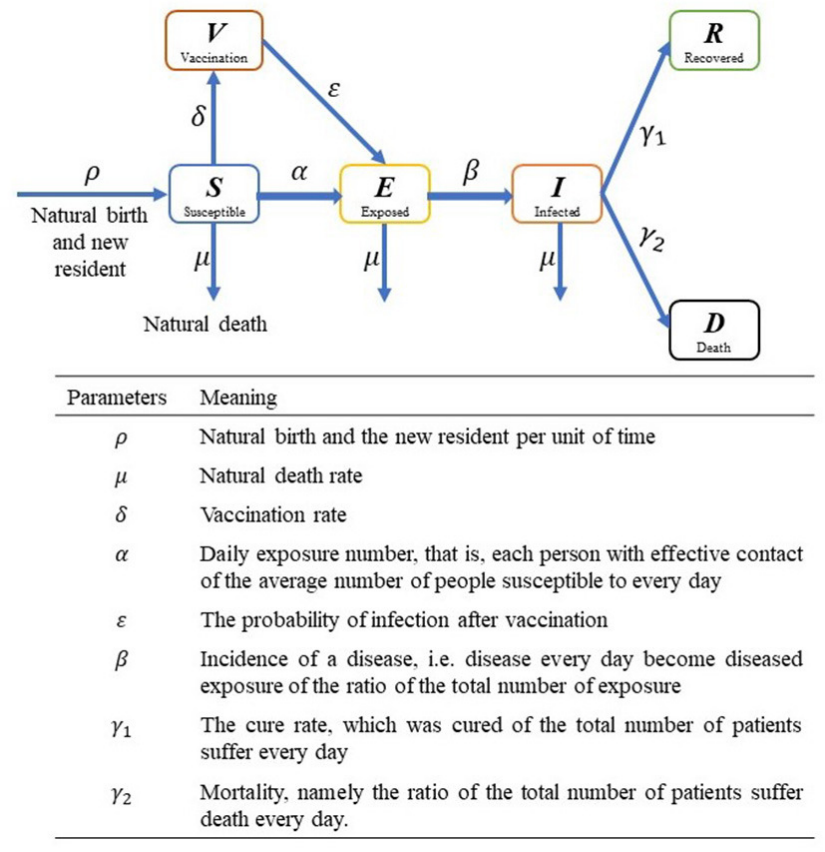
Illustration of compartmental model. S, E, I, R, D and V represent susceptible, exposed, infected, recovered, death and vaccination people, respectively.

After reading and summarizing the warehouse model and its variants proposed by existing researchers, the author made a graphical summary of all possible variants, roughly shown in Figure 3. A major flaw in these models, however, is their homogeneity mixing assumption, which assumes that individuals in the same compartment are in complete contact with each other and that they are equally likely to be infected by any one of the infected. It may be possible for a single small group, while if the population is larger, the internal spatial and social relationship structure tend to be more complex, and interpersonal interactions have a distinct individual orientation, such as family, friends and colleagues, or contact frequency between doctors and patients is significantly higher than the others, then this assumption would not work anymore. During the spread of COVID-19, it is found that a considerable number of infections have occurred in community or work unit, and a single infected person can infect a large number of people, which is the so-called “super spreader” phenomenon, which is significantly different from the transmission process of free environment (36).

***Metapopulation model***. The metapopulation model was originally derived from the field of bioecology and was mainly used to simulate the migration behavior of individuals between populations (78, 79), and were an extension of the compartmental model which is introduced into modeling the spread of infectious diseases (80–84). The main idea of the metapopulation model is to divide the population into several subgroups, namely the metapopulation, between which people can be allowed to travel, and within the metapopulation, the compartmental model can be used to simulate the transmission process of infectious diseases within the metapopulation. For example, the population of a country or region can be divided into cities, and the city can be further divided into communities.

Based on the Bats-Hosts-Reservoir-People transmission network, Chen et al. (37) constructed a transmission dynamics model of COVID-19 with metapopulations and multiple pathways, and calculated the transmission capacity of COVID-19 by stages in each province basically, thus realizing the phased transmission capacity assessment of COVID-19. Chinazzi et al. (38) applied the Global Epidemic and Mobility project (GLEaM) to analyze global subpopulations centered on transportation hubs, such as airports based on migration data from airports around the world. The subgroups are connected through population movement and individual travel (39). The SEIR model was constructed within each subpopulation to simulate the international spread of COVID-19, covering more than 3,200 subpopulations in about 200 countries and territories. They calculated the outbreak of unconstrained travel and compared the impact of travel restrictions on the epidemic with actual travel restrictions, and the epidemiological modeling results showed that for 90% of China, travel restrictions had an impact on the epidemiological trajectory unless transmission was reduced by more than 50% simultaneously within the community. It considers different transportation and interaction layers and distinguishes the mobility modeling from the dynamical process mediated by the human dynamics. This allows the integration of different processes of social contagion that are not necessarily of biological origin but occurs taking advantage of the individuals mobility such as information spreading, social behavior, etc. GLEaM has proved to be very flexible and we are working to make the GLEaM platform available to the scientific community at large.

The metapopulation model overcomes the shortcomings of the completely mixed hypothesis of compartmental model to a certain extent, but it is still a kind of macroscopic and relatively rough model that cannot describe the complex individual behavior patterns of infectious disease transmission, such as the movement rule of individuals in space and the behavior of responding to disease.

#### Micro-dynamic model

The individual-based model is a microscopic simulation model, which mainly includes CA and ABM based models. This modeling idea treats individuals in a population as cells or agents composed of a set of finite states and behavior rules. In general, the evolution behavior of such a complex infectious disease system consisting of virus, host and environment is simulated by defining rules such as individual response behavior to virus, individual movement behavior in space, and interaction behavior between individuals.

**Figure 4:**
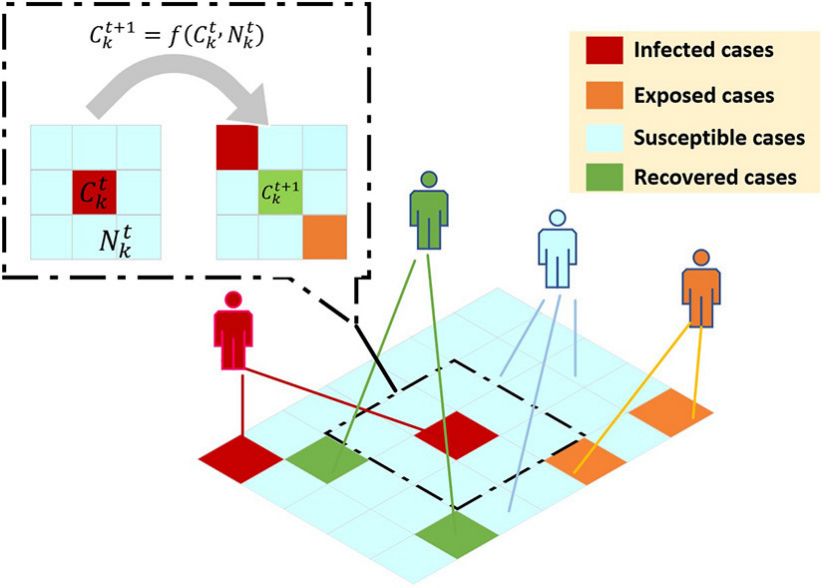
CA model to simulate the epidemic transmission based on virtual geographic grids. $C_{k}^{t+1}$ represents the cellular state of cell *k* at time *t*+1, $C_{k}^{t}$ represents the cellular state of cell *k* at time *t*, $N_{k}^{t}$ represents the neighborhood cellular state of cell *k* at time *t*, and *f* represents the transformation function of cell from the state at time *t* to the state at time *t*+1.

***CA model***. A typical CA model is defined on a grid. The grid of each point represents a cell in space and a finite state. Evolution rules apply to each cell and proceed simultaneously. The simple evolution of the cell unit simulates the complex dynamic evolution of the entire system. Traditional CA can be expressed by the following formula: $C_{k}^{t+1}=f\left(C_{k}^{t},N_{k}^{t}\right)$, where $C_{k}^{t+1}$represents the cellular state of cell *k* at time *t*+1, $C_{k}^{t}$ represents the cellular state of cell *k* at time *t*, $N_{k}^{t}$ represents the neighborhood cellular state of cell *k* at time *t*, and *f* represents the transformation function of cell from the state at time *t* to the state at time *t*+1. The main framework of CA model is illustrated in Figure 4.

CA has been used in many studies to model different aspects of epidemics. It has been widely used to model the disease spread of influenza and various vector-borne diseases, such as dengue (85–88). A neighborhood condition is an important aspect in the CA. The most used neighborhood conditions are (i) Neumann's neighborhood condition, (ii) Moore's neighborhood condition, (iii) Extended neighborhood condition, and (iv) Random interactions. Coupled with these neighborhood conditions, various models such as SEIR, SEIRS, SEIRD, and SEIRQD have been studied with the help of CA to model the spatial growth of epidemics (89, 90). Currently, CA has gained a lot of momentum in the studies of COVID-19 (40, 91, 92). Various advanced studies with Genetic algorithms and network models have been done for COVID-19 data (93–95). For example, Indian scholars used sequence evolutionary genetic algorithms to optimize the parameters of the CA model and simulate the development curve of COVID-19 (40, 41), this methodology can predict the varicella prevalence (with average relative error of 2–4%) in Belgium and Italy. The impact of different isolation systems on disease transmission was studied using Probabilistic Cellular Automaton (PCA) to simulate the spread of COVID-19 (42, 43), and the values of the basic reproduction number and the ratio and obtained in these simulations are similar to those found in real-world observations. However, these methods have two limitations. First, existing model do not take into account the effects of time correlation, and the prediction effects of the existing forms of continuous time series data are not ideal. None of the above models take into account the complex behavior of cells in the process of disease transmission, nor do they discuss the impact of various factors such as population movement on disease transmission, and fail to achieve the purpose of effective prediction. On the other hand, most of the existing CA-based infectious disease simulations are based on virtual geographic grids (simulation data), as is shown in Figure 4. Each small box or cell in the grid can be occupied by one person (44) rather than a real house or residential area. Although they can simulate the impact of policy changes on the spread of epidemics, they cannot, much less predict the spread of epidemics in actual areas.

Based on the data of confirmed COVID-19 cases, combined with cellular signaling data and spatial environmental data, spatial clustering analysis, factor analysis, and regression analysis were used to explore the spatio-temporal clustering characteristics of COVID-19 street scale in Chongqing, and analyze its influencing factors (96). Xia et al. combined with the spatio-temporal big data and epidemic dynamics models of multi-source cities in the Greater Bay Area (GBA), calculated the infection parameters (R0) of different communities in the city, corrected the SEIR model, constructed a suitable GBA and improved the regional model. Finally, the spread of COVID-19 in the GBA and the effectiveness of various epidemic prevention and control measures were evaluated and simulated (45). Xu et al. (97) collected and collated epidemiological data at the individual level, and identified them by geocoding to better monitor and predict the spread of infections at the spatial level. Ponce-de-Leon et al. (98) have developed a cross-referencing GIS that provides integrated datasets for managing, retrieving, visualizing and analyzing time series data from Spain's regularly updated population movement network and daily reports of COVID-19 cases. These models are based on CA or its variants to study the spread of infectious diseases at the spatial level. Current approaches for CA model transformation rule mining are micro-based and consider only the interactions between local meta-cellular units. However, in the real world, macroscopic factors, such as people wearing masks, keeping social distance, quarantine policies adopted by the government, vaccination and other measures, and medical resources in hospitals during an outbreak, will have a non-negligible impact on the transformation of microscopic meta-cellular units during the spread of an epidemic.

**Figure 5:**
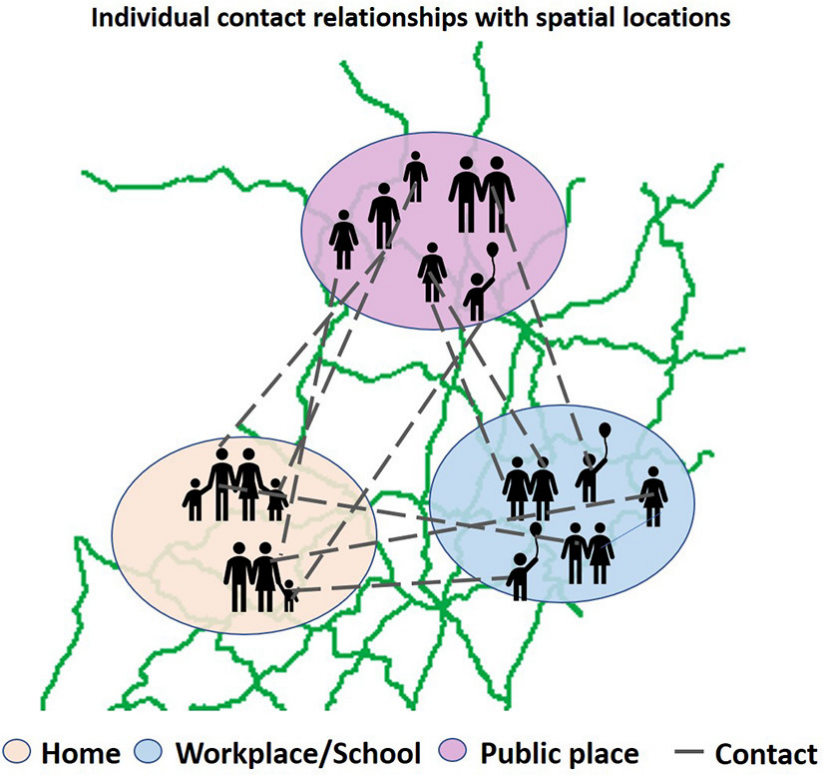
Agent based model to simulate epidemic transmission in real network. The broken line indicates interactions between agents in various places (home, workplace, school, public place).

***ABM model***. Compared with the CA model, the ABM model is an abstraction of real society, considering the movement of individuals in space and the social relationships between individuals. The ABM model is flexible, with individuals in the model autonomously changing their behaviors based on the environment and becoming more sensitive to government control. Moreover, the model is highly scalable and suitable for modeling and analyzing the spread of infectious diseases in different scenarios. Like we described in Figure 5, many researchers have used social contact networks to constrain the social activities and spatial distribution of individuals when modeling, and have achieved good performance (46, 47), the model estimated *R*0 = 2.0 in the early stages of the epidemic in China. The contributions to *R*0 included 46% from pre-symptomatic individuals (before showing symptoms), 38% from symptomatic individuals, 6% from asymptomatic individuals (who never show symptoms), and 10% from environmentally mediated transmission *via* contamination. Based on this method, the Los Alamos National Laboratory in the United States developed an EpiSimS simulation tool, which is suitable for simulating the spread of diseases in cities with a population of 1 million (99). EpiSimS generates virtual cities based on real demographic data to simulate the spread of diseases in cities. Eubank et al. (100) used EpiSimS to study the spread of the epidemic after terrorists used smallpox to attack Portland.

To response to COVID-19, better respond to ongoing epidemics and use computational models to predict the spread of infections and evaluate the impact of public health measures. Covid-tracing applications and research could track the risk of COVID-19 cases. For example, one study (101) aims to introduces a data-driven method to dynamically model infection risk of international ports of imported COVID-19 cases. The results show that the proposed method can track the risk of the imported COVID-19 of the main cruise ports worldwide. Hinch et al. (48) established OpenABM, an agent-based epidemic simulation model that can evaluate NPIs, including manual and digital contact tracing and vaccination procedures. It can simulate a population of 1 million in seconds per day, allowing parameter sweeping and inference based on formal statistical models, by performing a two-dimensional grid search across the prevalence at which a national lockdown was implemented (calibrated to 1.55%) and the reduction in daily contacts under lockdown (calibrated to 0.33 of pre-lockdown levels, it showed similar results to values reported from the first wave in the UK. Kerr et al. (49) build a Covasim model to predict pandemic trends, intervention options, and Estimated resource requirements. While the model correctly predicted the trend in cases, it underestimated the number of deaths, although the observations were still within the 80% forecast interval.

To make the predictions more accurate, Silva et al. (50) proposed a new SEIR (susceptible exposure-infection-recovery) agent-based COVID-ABS model, which aims to simulate pandemic dynamics using agent societies that simulate people, businesses, and governments, and seven simulation scenarios were conducted and it was concluded that a scenario with the fewest fatalities and the greatest economic impact could not be achieved in a lockdown scenario, and a combination of masks and partial isolation may be more realistic in terms of social cooperation. The results showed that COVID-ABS approach was capable to effectively simulate social intervention scenarios in line with the results presented in the literature. Another study (102) proposed an agent-based model and an implementation strategy for a technology-based contact tracing smart application, and explored the interaction between different adoption rates of contact tracing applications, different levels of detection capabilities, and behavioral factors role to assess the impact on the epidemic. This model can study specific factors between virus, host and environment at the micro level, and it is convenient to study specific factors researchers focus on. Its main disadvantage is low computational efficiency (38). To solve this problem, Wang et al. (51) supposed that each city is an agent with attributes such as urban population, number of infections, inter-city migration as links between nodes, thereby establishing a multi-agent urban network to simulate the inter-city communication process of COVID-19 among Chinese cities. The overall adapted city-based epidemic and mobility model (CEMM) they proposed has higher *R*^2^ and lower standard errors than CEMM, especially for cities outside Hubei Province, and the highest fitting accuracy (*R*^2^) reached 0.854 for all cities in China. Cuevas developed an agent based model to simulate the transmission risks in facilities, and proposed an agent-based model to simulate the spread of SARS-CoV-2 on a city scale. Researchers also proposed some new geospatial agent-based simulation model to explore the relation between the transmission of COVID-19 and intervention strategies (52, 53), the variation of each model parameter value by 20% had limited impact on outcome estimates (i.e., < 4,000 per 100,000 for incidence and 11 per 100,000 for mortality), suggesting the robustness of the results. And the developed ABM could help university managers to respond to current and future epidemics and plan effective responses to keep safe as many students as possible.

## Prospective research directions

**Figure 6:**
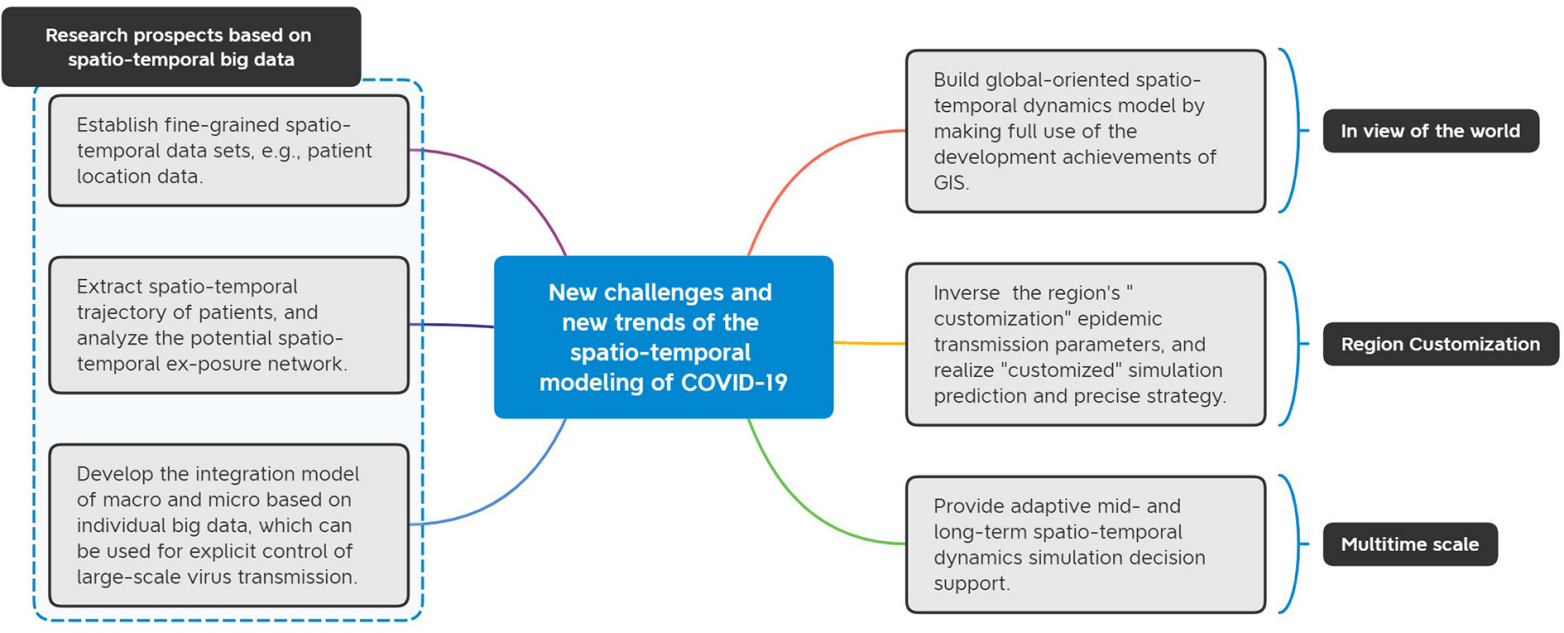
Prospects for future research on COVID-19 spatio-temporal dynamics models. The challenges and trends of building global-level models, making regional customized forecasts, providing multi-time-scale decision support, and developing model reproducibility based on spatiotemporal big data are prospected.

Due to the important role of spatio-temporal dynamics modeling in depicting the spread of infectious diseases and deepening the understanding of the characteristics of epidemic diseases, as well as the generation and support of spatio-temporal big data of infectious diseases, large number of COVID-19 dynamics models have been proposed, effectively guiding the actual work of epidemic prevention and control all over the world. It can be seen from the above research progress that the researchers used the compartmental model, metapopulation model and individual-based model to predict the epidemic trend, evaluate the NIPs and analyze the influencing factors of the epidemic. These studies have provided decision-making support for scientific response to the epidemic, but also raised some new challenges. We summarize the prospective future research direction below and illustrated in Figure 6.

(1) Establish a fine-grained spatio-temporal data set.

Data quality is a major limitation in modeling the spatio-temporal dynamics of infectious diseases and largely determines the effectiveness of modeling. Among the articles we analyzed, there were certain differences in the sources, acquisition and collation methods of COVID-19 data. Some researchers have pointed out that existing spatio-temporal big data of COVID-19 has some shortcomings, including statistical bias by test criteria, test methods and detection rates (103) and the omission of location-based case data (104). However, people tend to have more detailed expectations for dynamic modeling, such as the hope of simulating the development of the epidemic at the micro-scale and proposing precise intervention policies and suggestions, which makes the establishment of fine-grained spatial data integration an important prerequisite for accurate modeling of infectious diseases, especially location-based patient data. Therefore, for the public health sector, more fine-grained spatio-temporal data sets play a crucial and decisive role in dynamic modeling and are an important basis for dynamic modeling. With the development of cloud computing and crowdsourced data technology, it is possible to collect timely, trusted, and fine-grained large-scale epidemic datasets intelligently (105, 106).

(2) Extract the spatio-temporal trajectory of the patient.

The three elements of the spread of infectious diseases are the source of infection, the route of transmission and the susceptible population. Since the outbreak of COVID-19, some countries such as China have entered a stage of normalized epidemic prevention and control (107). Conventional epidemic prevention and control needs to quickly track the epidemic space, find the source of infection, cut off the transmission route, and control the epidemic with the least human and material resources. Therefore, how to build a spatio-temporal dynamics model, extract the spatio-temporal trajectory of patients in reverse, and analyze the potential spatio-temporal exposure network is another major challenge for infectious diseases dynamics modeling.

(3) Build a macro-micro fusion model based on individual big data.

As we summarized in this paper, macroscopic models are more suitable for simulating and describing the overall spread of the epidemic on a large scale, providing an effective reference for macroscopic decision-making, while ignoring individual behavioral responses to the disease. The micro model introduces the participation of individual behaviors to simulate the evolution of the on a small scale, but the calculation cost is high and the calculation parameters are complex. In the future, with the improvement of big data infrastructure and establishment of fine-grained individual big data (such as spatio-temporal location big data) (71, 108), the fact that some maps, like Amap, could be publicly published required taking special care with certain privacy concerns by reaching the optimal trade-off between public entitlement to being informed and the right to personal privacy. Therefore, it is possible to realize the integrated research of macro and micro models, and to study the effective interaction, mutual influence and restriction. Furthermore, based on visualization tools, this integrated model can be used to explicitly control the large-scale transmission of the virus.

(4) Establish a global-oriented spatio-temporal dynamics model.

This study reviews the application of a variety of dynamic models in infectious disease modeling of COVID-19, and argues that dynamic models in different disciplines have their own advantages and disadvantages, and are suitable for different modeling scales. The spatio-temporal dynamics model not only needs to consider the propagation characteristics of space, but also need to explore its spatio-temporal development law. In the current era of big data, the spatio-temporal changes in information collection methods and fine-grained data, as well as the development of machine learning and data driven, make it possible to build a spatio-temporal infectious disease dynamics model that is interdisciplinary and oriented to a global perspective (109). Future research on the dynamics model of infectious diseases should consider the complexity requirements of the transmission and prevention and control processes. On the basis of strengthening the rapid recognition and prevention and control of infectious diseases, promoting the real-time sharing of epidemic information and exploring the combined application of multiple models, it is necessary to make full use of the development achievements of GIS (24), computer science and intelligent frontier technologies (27, 110). Thus, to provide theoretical support and data support for public health decision-making and policy makers. As can be seen from the COVID-19 pandemic, infectious diseases in today's world are a serious challenge. Studying the COVID-19 pandemic as a global issue, developing a system dynamic model to study global issues, and conducting causal analysis will help countries control the epidemic.

(5) Realize “customized” simulation prediction and precise strategy.

Epidemic prediction is one of the most important methods of epidemic prevention and control. Affected by the national conditions of various countries and the actual epidemic prevention policies of different regions, the spread of the epidemic in different regions and cities is also different. The spatio-temporal dynamic integrated model is based on historical epidemic data, inverts a specific area, and obtains the “customization” epidemic transmission parameters in the area, in order to simulate and predict the epidemic situation after fighting against secondary or other types of infectious diseases in the area. This requires that compared with current machine learning methods that focus on short-term epidemic scale for accurate prediction, deep learning methods based on spatio-temporal characterization should focus more on extrapolating the medium- and long-term trends of disease development, simulating and evaluating the effects of different interventions, and revealing some hidden features of the disease transmission process, that is, not to accurately predict the future scale of infection as a single modeling purpose, but more so as a key planning method that can be used for intervention strategy deployment and regulation decisions in epidemics. The future research direction of epidemic spatio-temporal dynamics model should be to introduce a multi-layer and multi-scale integrated model of epidemic spatio-temporal dynamics with big data and intelligent computing, and to realize “customized” simulation prediction and precise strategy by strengthening the research and development of meso-dynamic model (27).

(6) Provide adaptive mid- and long-term spatio-temporal dynamics simulation decision support.

Furthermore, in the face of so many infectious disease models, there is urgent need to develop widely applicable and public modeling tools and platforms, especially spatially explicit modeling tools based on GIS (71). This tool can not only provide for researchers with a verifiable scientific research platform, but also provide a convenient scientific model for public health departments and policy makers, become an auxiliary means of daily decision-making, and promote the process of public health scientific decision-making. Therefore, an adaptive mid- and long-term (from months to year) spatio-temporal dynamics simulation decision support model combined with medical conclusions will be a technical problem to be tackled in the next stage.

(7) Improve the reusability of spatio-temporal propagation model.

Although some studies have published source code, the exact specification of the methods and parameters required to reproduce these results is opaque and thus the results are not reproducible (23, 111). Studying the spatio-temporal transmission model of COVID-19 infectious diseases is not only to provide suggestions for the modeling of the spread of COVID-19, but more importantly, to provide scientific and technological support and model reference for other major public health emergencies, and to help the simulation and prediction after the outbreak of other types of infectious diseases. Therefore, it is necessary to improve the reusability of the spatio-temporal propagation model.

## Discussion and conclusion

This study aims to reveal the modeling methods and trends of COVID-19 spatio-temporal transmission from the perspective of literature induction, and divide the spatio-temporal dynamic modeling models of COVID-19 into macro modeling and micro modeling at the research scale. On the basis of previous reviews, our current research further expand on the previous review and further integrate spatial models of COVID-19, focusing on analyzing spatio-temporal modeling models related to COVID-19. Our innovation is to fill in the gaps in the spatio-temporal dynamic modeling of COVID-19 review studies, review the development of this field, and recommend further research to enable more people to work together in public health prevention and control, and help to provide targeted responses to COVID-19 as soon as possible.

Geographical Information Science (GIScience) must be considered in promoting spatio-temporal dynamic modeling, whether it is macro model or micro model. GIScience provides a new tool to promote public health, helps us to understand the world around us by capturing, quantifying and deploying the wealth of geographical information available. Traditional geographic methods have many limitations in this regard, as people move a lot in their daily lives and interact with dynamic environments in complex ways. With advanced geospatial technologies such as mobile tracking and sensing and the GIScience method, we can know when people are affected by what environment, and accurately measure the degree of environmental impact on people's health and the spread of disease. GIScience has been used to do a lot of research on COVID-19-related research, such as studying the impact of human movement on the spread of COVID-19 (112), identifying the spatio-temporal patterns of COVID-19 risk and its association with different built environment characteristics (113–115), spatial analysis of the impact of urban geometry and sociodemographic characteristics on COVID-19 (116). Some study (115) explores the spatial patterns of COVID-19 transmission and its key determinants could provide a deeper understanding of the evolution of the COVID-19 pandemic, another research (117) successfully developed a vector field approach to evaluate the environmental exposure at the population level. However, with its focus on crunching vast amounts of data, it can sometimes lose track of the human factor: how people interact with their environment at an individual level, and what that means for them. This is what GIScience should focus on when it is combined with public health in the future. In the reflection of this review, we believe that in order to respond to the COVID-19 global pandemic, positive measures should be taken from an interdisciplinary perspective, through international solidarity and cooperation, and from a global perspective (118, 119). However, it turns out that although this starting point is good, it will cause certain contradictions at the political level (120).

Our research also has some limitations. First, we mostly focus on modeling the spread of the epidemic at present, and post-epidemic spatio-temporal modeling is also a very important aspect (121). Second, with the development of information technology, geospatial data is growing by at least 20% per year (122) and accessibility is increasing year by year. In the face of these data, how to establish a GIS online application that is convenient for public tasks is also an important way to show the results of the dynamic modeling of spatio-temporal propagation, due to the difficulty of data acquisition, we have not made a unified induction here. Also, in our spatio-temporal dynamic modeling study of COVID-19, we only used Google Scholar and the WOS database. Our search period was 32 months (January 2020–August 2022) and articles published thereafter are not included in this review. In addition, we only consider articles published in English. Therefore, there is an article that, while it satisfies other eligibility criteria, has had to be excluded due to language reasons. According to the performance of major infectious disease in the past, the spread of epidemics in time and space has obvious differences due to different environmental factors (123–125), medical conditions (23), government decisions (126, 127), social systems (128, 129), etc. The impact analysis of the model is also an important aspect. In the macro- and micro-models reviewed in this paper, although they are covered to a certain extent, they are not comprehensive enough.

To the best of our knowledge, this is one of the first systematic reviews of the spatio-temporal modeling of COVID-19 related research. Our work does not only provide an overview of how macro-dynamic and micro-dynamic models was used so far but also provides pointers on how those method and GIScience could be more efficiently used in COVID-19-related works and other public health issues in the coming days. Our study also sheds light on analyzing the progressive relationship between the models and their strengths and weaknesses. The inductive study of these models and results analysis have important implications for our understanding of the transmission dynamics of the COVID-19 pandemic and for developing prevention and control strategies to contain the spread and progression of the disease. In the early stage of the COVID-19 outbreak, most dynamics modeling studies were based on public health data and global flight data to simulate the spread and change of the epidemic on a global and national scale from a macro-modeling perspective. Although this simulation can give a rough prediction of epidemic development in a short time, it cannot achieve accurate simulation on a small scale. Therefore, a model based on population division was proposed to achieve more accurate macro modeling. With the development of computer technology and the disclosure of mobile phone signaling data by major operators, researchers have carried out some micro studies based on these fine-grained data. Individual-based modeling provides the possibility to evaluate the dynamic modeling of social contact and social relationship structure on the spread of infectious diseases.

The dynamic modeling method can better reflect the epidemic law from the transmission mechanism of the disease and can consider the global status in the epidemic process. The methods of infectious disease dynamics, biostatistics and computer simulation are combined to deepen the understanding of the epidemic law of infectious diseases, and make the established model and prevention strategy more consistent with the reality. Meanwhile, we have noticed that each model has its own advantages and disadvantages. Without the premise of practical application, we cannot say that the more complex the model will perform better. Because simple models often contain fewer parameters, and complex models often contain more parameters, the uncertainty of the model is increased from the determination of parameters. In addition, micro-models are not necessarily better than macro-models, because micro-models often require large computing resources, which is also a non-negligible challenge for modeling. The mechanism and parameter selection of micro and macro models need to be further studied in our next research.

## Author contributions

XZ and HL: conceptualization and further analysis. PW, XZ, and HL: systematic literature review, and writing—review and editing. PW: in-depth analysis and writing—original draft preparation. XZ: useful comments. XZ and PW: funding acquisition. All authors have read and agreed to the published version of the manuscript.

## Funding

This research was funded by the Fundamental Research Funds for the Central Universities, grant number 2652020002 and 2021 Graduate Innovation Fund Project of China University of Geosciences, Beijing, grant number ZY2021YC010.

## Conflict of interest

The authors declare that the research was conducted in the absence of any commercial or financial relationships that could be construed as a potential conflict of interest.

## Publisher's note

All claims expressed in this article are solely those of the authors and do not necessarily represent those of their affiliated organizations, or those of the publisher, the editors and the reviewers. Any product that may be evaluated in this article, or claim that may be made by its manufacturer, is not guaranteed or endorsed by the publisher.

## References

[1] Glass GEJE. Update: spatial aspects of epidemiology: the interface with medical geography. Epidemiol Rev. (2000) 22:136–9. 10.1093/oxfordjournals.epirev.a018010 10939019

[2] Hai-Feng Z Michael S Xin-Chu F Bing-Hong WJCPB. Dynamical behaviour of an epidemic on complex networks with population mobility. Chin Phys B. (2009) 18:3639. 10.1088/1674-1056/18/9/00621130777

[3] Zhang Y Zhang A Wang J. Exploring the roles of high-speed train, air and coach services in the spread of COVID-19 in China. Transp Policy. (2020) 94:34–42. 10.1016/j.tranpol.2020.05.012 32501380 PMC7248624

[4] Jia JS Lu X Yuan Y Xu G Jia J Christakis NA. Population flow drives spatio-temporal distribution of COVID-19 in China. Nature. (2020) 582:389–94. 10.1038/s41586-020-2284-y 32349120

[5] Hou X Gao S Li Q Kang Y Chen N Chen K. Intracounty modeling of COVID-19 infection with human mobility: assessing spatial heterogeneity with business traffic, age, and race. Proc Natl Acad Sci U S A. (2021) 118:e2020524118. 10.1073/pnas.2020524118 34049993 PMC8214685

[6] Pradas-Velasco R Antoñanzas-Villar F Martínez-Zárate MPJP. Dynamic modelling of infectious diseases. Pharmacoeconomics. (2008) 26:45–56. 10.2165/00019053-200826010-00005 18088158

[7] Heesterbeek H Anderson RM Andreasen V Bansal S De Angelis D Dye C., Modeling infectious disease dynamics in the complex landscape of global health. Science. (2015) 347:aaa4339. 10.1126/science.aaa4339 25766240 PMC4445966

[8] Daw MA El-Bouzedi AH Ahmed MO. The epidemiological and spatiotemporal characteristics of the 2019 novel coronavirus disease (COVID-19) in Libya. Front Public Health. (2021) 9:8. 10.3389/fpubh.2021.628211 34195168 PMC8236517

[9] Colizza V Barthélemy M Barrat A Vespignani AJCRB. Epidemic modeling in complex realities. C R Biol. (2007) 330:364–74. 10.1016/j.crvi.2007.02.014 17502293

[10] Cliff OM Harding N Piraveenan M Erten EY Gambhir M Prokopenko M. Investigating spatiotemporal dynamics and synchrony of influenza epidemics in Australia: an agent-based modelling approach. Simul Model Pract Theory. (2018) 87:412–31. 10.1016/j.simpat.2018.07.005

[11] Kissler SM Tedijanto C Goldstein E Grad YH Lipsitch M. Projecting the transmission dynamics of SARS-CoV-2 through the postpandemic period. Science. (2020) 368:860–8. 10.1126/science.abb5793 32291278 PMC7164482

[12] Zheng N Du S Wang J Zhang H Cui W Kang Z. Predicting COVID-19 in China using hybrid AI model. IEEE Trans Cybern. (2020) 50:2891–904. 10.1109/TCYB.2020.2990162 32396126

[13] Ala'raj M Majdalawieh M Nizamuddin NJIDM. Modeling and forecasting of COVID-19 using a hybrid dynamic model based on SEIRD with ARIMA corrections. Infect Dis Model. (2021) 6:98–111. 10.1016/j.idm.2020.11.007 33294749 PMC7713640

[14] Roda WC Varughese MB Han D Li MYJIDM. Why is it difficult to accurately predict the COVID-19 epidemic? Infect Dis Model. (2020) 5:271–81. 10.1016/j.idm.2020.03.001 32289100 PMC7104073

[15] Callaway EJN. Fast-spreading COVID variant can elude immune responses. Nature. (2021) 2021:500–1. 10.1038/d41586-021-00121-z 33479534

[16] McKibbin W Fernando RJETC. The economic impact of COVID-19. Sylwan. (2020) 45:1162. 10.2139/ssrn.3547729

[17] You S Wang H Zhang M Song H Xu X Lai YJH. Assessment of monthly economic losses in Wuhan under the lockdown against COVID-19. Hum Soc Sci Commun. (2020) 7:1–12. 10.1057/s41599-020-00545-4

[18] Li Z Li X Porter D Zhang J Weissman S. Monitoring the spatial spread of COVID-19 and effectiveness of control measures through human movement data: proposal for a predictive model using big data analytics. JMIR Res Protoc. (2020) 9:1–10. 10.2196/preprints.24432 33301418 PMC7752182

[19] Mourad N Habib A Tharwat A. Appraising healthcare systems' efficiency in facing COVID-19 through data envelopment analysis. Decis Sci Lett. (2021) 10:301–10. 10.5267/j.dsl.2021.2.007

[20] Elias C Sekri A Leblanc P Cucherat M Vanhems P. The incubation period of COVID-19: a meta-analysis. Int J Infect Dis. (2021) 104:708–10. 10.1016/j.ijid.2021.01.069 33548553 PMC7857041

[21] Choi S Ki M. Estimating the reproductive number and the outbreak size of COVID-19 in Korea. Epidemiol Health Place. (2020) 42:e2020011. 10.4178/epih.e2020011 32164053 PMC7285447

[22] Baud D Qi X Nielsen-Saines K Musso D Pomar L Favre G. Real estimates of mortality following COVID-19 infection. Lancet Infect Dis. (2020) 20:773. 10.1016/S1473-3099(20)30195-X 32171390 PMC7118515

[23] Huang B Wang J Cai J Yao S Chan PKS Tam TH. Integrated vaccination and physical distancing interventions to prevent future COVID-19 waves in Chinese cities. Nat Hum Behav. (2021) 5:695–705. 10.1038/s41562-021-01063-2 33603201

[24] Franch-Pardo I Napoletano BM Rosete-Verges F Billa L. Spatial analysis and GIS in the study of COVID-19. A review. Sci Total Environ. (2020) 739:140033. 10.1016/j.scitotenv.2020.140033 32534320 PMC7832930

[25] Fatima M O'Keefe KJ Wei W Arshad S Gruebner O. Geospatial analysis of COVID-19: a scoping review. Int J Environ Res Public Health. (2021) 18:2336. 10.3390/ijerph18052336 33673545 PMC7956835

[26] Wang P Zheng X Li J Zhu B. Prediction of epidemic trends in COVID-19 with logistic model and machine learning technics. Chaos Solitons Fract. (2020) 139:110058. 10.1016/j.chaos.2020.110058 32834611 PMC7328553

[27] Yesilkanat CM. Spatio-temporal estimation of the daily cases of COVID-19 in worldwide using random forest machine learning algorithm. Chaos Solitons Fract. (2020) 140:8. 10.1016/j.chaos.2020.110210 32843823 PMC7439995

[28] Jin BA Ji JW Yang WH Yao ZQ Huang DD Xu C. Analysis on the spatio-temporal characteristics of COVID-19 in mainland China. Process Saf Environ Protect. (2021) 152:291–303. 10.1016/j.psep.2021.06.004 34121818 PMC8183012

[29] Karadayi Y Aydin MN Ogrenci AS. Unsupervised anomaly detection in multivariate spatio-temporal data using deep learning: early detection of COVID-19 outbreak in Italy. IEEE Access. (2020) 8:164155–77. 10.1109/ACCESS.2020.3022366 34931155 PMC8668158

[30] He S Peng Y Sun K. SEIR modeling of the COVID-19 and its dynamics. Nonlinear Dyn. (2020) 101:1667–80. 10.1007/s11071-020-05743-y 32836803 PMC7301771

[31] Team IC-F. Modeling COVID-19 scenarios for the United States. Nat Med. (2021) 27:94–105. 10.1101/2020.07.12.20151191v1 33097835 PMC7806509

[32] Cui Q Hu Z Li Y Han J Teng Z Qian J. Dynamic variations of the COVID-19 disease at different quarantine strategies in Wuhan and mainland China. J Infect Public Health. (2020) 13:849–55. 10.1016/j.jiph.2020.05.014 32493669 PMC7242968

[33] Liu FL Wang J Liu JW Li Y Liu DG Tong JL. Predicting and analyzing the COVID-19 epidemic in China: based on SEIRD, LSTM and GWR models. PLoS ONE. (2020) 15:22. 10.1371/journal.pone.0238280 32853285 PMC7451659

[34] Godio A Pace F Vergnano A Health P. SEIR modeling of the Italian epidemic of SARS-CoV-2 using computational swarm intelligence. Int J Environ Res Public Health. (2020) 17:3535. 10.3390/ijerph17103535 32443640 PMC7277829

[35] Yang Z Zeng Z Wang K Wong S-S Liang W Zanin M. Modified SEIR and AI prediction of the epidemics trend of COVID-19 in China under public health interventions. J Thorac Dis. (2020) 12:165–74. 10.21037/jtd.2020.02.64 32274081 PMC7139011

[36] Wang L Didelot X Yang J Wong G Shi Y Liu W. Inference of person-to-person transmission of COVID-19 reveals hidden super-spreading events during the early outbreak phase. Nat Commun. (2020) 11:1–6. 10.1038/s41467-020-18836-4 33024095 PMC7538999

[37] Chen T-M Rui J Wang Q-P Zhao Z-Y Cui J-A Yin L. A mathematical model for simulating the phase-based transmissibility of a novel coronavirus. Infect Dis Poverty. (2020) 9:1–8. 10.1186/s40249-020-00640-3 32111262 PMC7047374

[38] Balcan D Goncalves B Hu H Ramasco JJ Colizza V Vespignani A. Modeling the spatial spread of infectious diseases: the GLobal epidemic and mobility computational model. J Comput Sci. (2010) 1:132–45. 10.1016/j.jocs.2010.07.002 21415939 PMC3056392

[39] Chinazzi M Davis JT Ajelli M Gioannini C Litvinova M Merler S. The effect of travel restrictions on the spread of the 2019 novel coronavirus (COVID-19) outbreak. Sicence. (2020) 368:395–400. 10.1126/science.aba9757 32144116 PMC7164386

[40] Ghosh S Bhattacharya S. A data-driven understanding of COVID-19 dynamics using sequential genetic algorithm based probabilistic cellular automata. Appl Soft Comput. (2020) 96:106692. 10.1016/j.asoc.2020.106692 32904415 PMC7455552

[41] Monteiro LHA Gandini D Schimit PHT. The influence of immune individuals in disease spread evaluated by cellular automaton and genetic algorithm. Comput Methods Prog Biomed. (2020) 196:105707. 10.1016/j.cmpb.2020.105707 32853857 PMC7434376

[42] Monteiro L. Fanti v, Tessaro AJEC. On the spread of SARS-CoV-2 under quarantine: a study based on probabilistic cellular automaton. Ecol Compl. (2020) 44:100879. 10.1016/j.ecocom.2020.100879

[43] Medrek M Pastuszak Z. Numerical simulation of the novel coronavirus spreading. Exp Syst Appl. (2021) 166:114109. 10.1016/j.eswa.2020.114109 33078047 PMC7557303

[44] Ghosh S Bhattacharya SJSCS. Computational model on COVID-19 pandemic using probabilistic cellular automata. SN Comput Sci. (2021) 2:1–10. 10.1007/s42979-021-00619-3 33907736 PMC8061453

[45] Jizhe X Ying Z Zhen L Fan L Yang Y Tao C. COVID-19 risk assessment driven by urban spatiotemporal big data: a case study of Guangdong-Hong Kong-Macao greater bay area. Acta Geodaetica et Cartographica Sinica. (2020) 49:671. 10.11947/j.AGCS.2020.20200080

[46] Ferretti L Wymant C Kendall M Zhao L Nurtay A Abeler-Dörner L., Quantifying SARS-CoV-2 transmission suggests epidemic control with digital contact tracing. Science. (2020) 368:eabb6936 10.1126/science.abb6936 32234805 PMC7164555

[47] Keskinocak P Oruc BE Baxter A Asplund J Serban N. The impact of social distancing on COVID19 spread: State of Georgia case study. PLoS ONE. (2020) 15:e0239798. 10.1371/journal.pone.0239798 33045008 PMC7549801

[48] Hinch R Probert WJ Nurtay A Kendall M Wymant C Mall H., OpenABM-Covid19—an agent-based model for non-pharmaceutical interventions against COVID-19 including contact tracing. PLoS Comput Biol. (2021) 17:e1009146. 10.1371/journal.pcbi.1009146 34252083 PMC8328312

[49] Kerr CC Stuart RM Mistry D Abeysuriya RG Rosenfeld K Hart GR. Covasim: an agent-based model of COVID-19 dynamics and interventions. PLoS Comput Biol. (2021) 17:e1009149. 10.1371/journal.pcbi.1009149 34310589 PMC8341708

[50] Silva PC Batista PV Lima HS Alves MA Guimarães FG Silva RCJC. COVID-ABS: an agent-based model of COVID-19 epidemic to simulate health and economic effects of social distancing interventions. Chaos Solitons Fract. (2020) 139:110088. 10.1016/j.chaos.2020.110088 32834624 PMC7340090

[51] Wei Y Wang J Song W Xiu C Ma L Pei T. Spread of COVID-19 in China: analysis from a city-based epidemic and mobility model. Cities. (2021) 110:103010. 10.1016/j.cities.2020.103010 33162634 PMC7598765

[52] Castro DA Ford A. 3D agent-based model of pedestrian movements for simulating COVID-19 transmission in University students. ISPRS Int J Geo-Inform. (2021) 10:44. 10.3390/ijgi10080509

[53] Hoertel N Blachier M Blanco C Olfson M Massetti M Limosin F., Facing the COVID-19 epidemic in NYC: a stochastic agent-based model of various intervention strategies. MedRxiv. (2020). 10.1101/2020.04.23.20076885 32511467 PMC7255787

[54] Dong E Du H Gardner L. An interactive web-based dashboard to track COVID-19 in real time. Lancet Infect Dis. (2020) 20:533–4. 10.1016/S1473-3099(20)30120-1 32087114 PMC7159018

[55] Nouvellet P Bhatia S Cori A Ainslie KE Baguelin M Bhatt S. Reduction in mobility and COVID-19 transmission. Nat Commun. (2021) 12:1–9. 10.1038/s41467-021-21358-2 33597546 PMC7889876

[56] Assadian O Golling M Kruger CM Leaper D Mutters NT. R Both, et al., Surgical site infections: guidance for elective surgery during the SARS-CoV-2 pandemic: international recommendations and clinical experience. J Hosp Infect. (2021) 111:189–99. 10.1016/j.jhin.2021.02.011 33600892 PMC7883712

[57] Wissel BD Van Camp P Kouril M Weis C Glauser TA White PS. An interactive online dashboard for tracking COVID-19 in US counties, cities, and states in real time. J Am Med Inform Assoc. (2020) 27:1121–5. 10.1093/jamia/ocaa071 32333753 PMC7188179

[58] Gao S Rao J Kang Y Liang Y Kruse J Dopfer D. Association of mobile phone location data indications of travel and stay-at-home mandates with COVID-19 infection rates in the US. JAMA Netw Open. (2020) 3:e2020485. 10.1001/jamanetworkopen.2020.20485 32897373 PMC7489834

[59] Tyrovolas S Gine-Vazquez I Fernandez D Morena M Koyanagi A Janko M. Estimating the COVID-19 spread through real-time population mobility patterns: surveillance in low- and middle-income countries. J Med Internet Res. (2021) 23:16. 10.2196/22999 33950850 PMC8204939

[60] Laughner JL Neu JL Schimel D Wennberg PO Barsanti K Bowman KW. Societal shifts due to COVID-19 reveal large-scale complexities and feedbacks between atmospheric chemistry and climate change. Proc Natl Acad Sci U S A. (2021) 118:12. 10.1002/essoar.10506081.3 34753820 PMC8609622

[61] Li Z Li X Porter D Zhang J Jiang Y Olatosi B. Monitoring the spatial spread of COVID-19 and effectiveness of control measures through human movement data: proposal for a predictive model using big data analytics. JMIR Res Protoc. (2020) 9:e24432. 10.2196/24432 33301418 PMC7752182

[62] Subramanian S Kumar A. Increases in COVID-19 are unrelated to levels of vaccination across 68 countries and 2947 counties in the United States. Eur J Epidemiol. (2021) 36:1237–40. 10.1007/s10654-021-00808-7 34591202 PMC8481107

[63] Pozo-Martin F Weishaar H Cristea F Hanefeld J Bahr T Schaade L. The impact of non-pharmaceutical interventions on COVID-19 epidemic growth in the 37 OECD member states. Eur J Epidemiol. (2021) 2021:1–12. 10.1007/s10654-021-00766-0 34114189 PMC8192111

[64] Chew NW Cheong C Kong G Phua K Ngiam JN Tan BY. An Asia-Pacific study on healthcare workers' perceptions of, and willingness to receive, the COVID-19 vaccination. Int J Infect Dis. (2021) 106:52–60. 10.1016/j.ijid.2021.03.069 33781902 PMC7997703

[65] Desvars-Larrive A Dervic E Haug N Niederkrotenthaler T Chen J Di Natale A. A structured open dataset of government interventions in response to COVID-19. Sci Data. (2020) 7:1–9. 10.1038/s41597-020-00609-9 32855430 PMC7452888

[66] Huang R Liu M Ding Y. Spatial-temporal distribution of COVID-19 in China and its prediction: a data-driven modeling analysis. J Infect Develop Count. (2020) 14:246–53. 10.3855/jidc.12585 32235084

[67] Fang Y Nie Y Penny M. Transmission dynamics of the COVID-19 outbreak and effectiveness of government interventions: a data-driven analysis. J Med Virol. (2020) 92:645–59. 10.1002/jmv.25750 32141624 PMC7228381

[68] Anastassopoulou C Russo L Tsakris A Siettos CJP. Data-based analysis, modelling and forecasting of the COVID-19 outbreak. PLoS ONE. (2020) 15:e0230405. 10.1371/journal.pone.0230405 32231374 PMC7108749

[69] Zhou Y Wang L Zhang L Shi L Yang K He J. A Spatiotemporal Epidemiological Prediction Model to Inform County-Level COVID-19 Risk in the United States. Cambridge, MA: Harvard Data Science Review (2020). 10.1162/99608f92.79e1f45e

[70] Iranmanesh A Atun RA. Reading the changing dynamic of urban social distances during the COVID-19 pandemic via Twitter. Eur Soc. (2021) 23:S872–86. 10.1080/14616696.2020.1846066

[71] Zhou C Su F Pei T Zhang A Du Y Luo B. COVID-19: challenges to GIS with big data. Geogr Sustain. (2020) 1:77–87. 10.1016/j.geosus.2020.03.005

[72] Anderson RM May RM. Infectious Diseases of Humans: Dynamics and Control.Oxford: Oxford University Press (1992).

[73] Kermack WO McKendrick AG. A contribution to the mathematical theory of epidemics. Proc R Soc Lond Ser A Contain Papers Math Phys Charact. (1927) 115:700–21.

[74] Satsuma J Willox R Ramani A Grammaticos B Carstea A. Applications, extending the SIR epidemic model. Phys A Stat Mech Appl. (2004) 336:369–75. 10.1016/j.physa.2003.12.035

[75] Ng TW Turinici G Danchin A. A double epidemic model for the SARS propagation. BMC Infect Dis. (2003) 3:1–16. 10.1186/1471-2334-3-19 12964944 PMC222908

[76] Werner PA Kesik-Brodacka M Nowak K Olszewski R Kaleta M Liebers DT. Modeling the spatial and temporal spread of COVID-19 in Poland based on a spatial interaction model. ISPRS Int J Geo-Inform. (2022) 11:195. 10.3390/ijgi11030195

[77] de Lima CL da Silva CC da Silva ACG Silva EL Marques GS de Araujo LJB. COVID-SGIS: a smart tool for dynamic monitoring and temporal forecasting of Covid-19. Front Public Health. (2020) 8:21. 10.3389/fpubh.2020.580815 33282815 PMC7705350

[78] Fulford G Roberts M Heesterbeek J. The metapopulation dynamics of an infectious disease: tuberculosis in possums. Theor Popul Biol. (2002) 61:15–29. 10.1006/tpbi.2001.1553 11895380

[79] Vergu E Busson H Ezanno P. Impact of the infection period distribution on the epidemic spread in a metapopulation model. PLoS ONE. (2010) 5:e9371. 10.1371/journal.pone.0009371 20195473 PMC2829081

[80] Hanski IJN. Metapopulation dynamics. Nature. (1998) 396:41–9.

[81] Keeling MJ Gilligan CAJN. Metapopulation dynamics of bubonic plague. Nature. (2000) 407:903–6. 10.1038/35038073 11057668

[82] Colizza V Pastor-Satorras R Vespignani A. Reaction–diffusion processes and metapopulation models in heterogeneous networks. Nat Phys. (2007) 3:276–82. 10.1038/nphys560

[83] Vazquez A. Epidemic outbreaks on structured populations. J Theor Biol. (2007) 245:125–9. 10.1016/j.jtbi.2006.09.018 17097683

[84] Watts DJ Muhamad R Medina DC Dodds P. Multiscale, resurgent epidemics in a hierarchical metapopulation model. Proc Nat Acad Sci. (2005) 102:11157–62. 10.1073/pnas.0501226102 16055564 PMC1183543

[85] Holko A Mȩdrek M Pastuszak Z Phusavat K. Epidemiological modeling with a population density map-based cellular automata simulation system. Exp Syst Appl. (2016) 48:1–8. 10.1016/j.eswa.2015.08.018

[86] Athithan S Shukla VP Biradar SR. Epidemic spread modeling with time variant infective population using pushdown cellular automata. J Comput Environ Sci. (2014) 2014:10. 10.1155/2014/769064

[87] Bin S Sun G Chen C-C. Spread of infectious disease modeling and analysis of different factors on spread of infectious disease based on cellular automata. Int J Environ Res Public Health. (2019) 16:4683. 10.3390/ijerph16234683 31775236 PMC6926909

[88] Ortigoza G Brauer F Neri I. Modelling and simulating chikungunya spread with an unstructured triangular cellular automata. Infect Dis Model. (2020) 5:197–220. 10.1016/j.idm.2019.12.005 32021947 PMC6993010

[89] Gagliardi HF Alves D. Small-world effect in epidemics using cellular automata. Math Popul Stud. (2010) 17:79–90. 10.1080/08898481003689486

[90] Athithan S Shukla VP Biradar SR. Dynamic cellular automata based epidemic spread model for population in patches with movement. J Comput Environ Sci. (2014) 2014:8. 10.1155/2014/518053

[91] Schimit PHT. A model based on cellular automata to estimate the social isolation impact on COVID-19 spreading in Brazil. Comput Methods Prog Biomed. (2020) 2020:105832. 10.1016/j.cmpb.2020.105832 33213971 PMC7836885

[92] Jithesh PK. A model based on cellular automata for investigating the impact of lockdown, migration and vaccination on COVID-19 dynamics. Comput Methods Prog Biomed. (2021) 211:10. 10.1016/j.cmpb.2021.106402 34530391 PMC8423711

[93] Fraga LM de Oliveira GM Martins LG. “Multistage evolutionary strategies for adjusting a cellular automata-based epidemiological model,” in *Proceedings of the 2021 IEEE Congress on Evolutionary Computation (CEC)* (Piscataway, NJ: IEEE) (2021). 10.1109/CEC45853.2021.9504738

[94] Lima L Atman A. Impact of mobility restriction in COVID-19 superspreading events using agent-based model. PLoS ONE. (2021) 16:e0248708. 10.1371/journal.pone.0248708 33735290 PMC7971565

[95] Sahasranaman A Jensen HJ. Spread of COVID-19 in urban neighbourhoods and slums of the developing world. J R Soc Interf. (2021) 18:20200599. 10.1098/rsif.2020.0599 33468021 PMC7879756

[96] He S Chen S Kong L Liu W. Analysis of risk perceptions and related factors concerning COVID-19 epidemic in Chongqing, China. J Commun Health. (2021) 46:278–85. 10.1007/s10900-020-00870-4 32592160 PMC7318903

[97] Xu B Gutierrez B Mekaru S Sewalk K Goodwin L Loskill A. Epidemiological data from the COVID-19 outbreak, real-time case information. Sci Data. (2020) 7:1–6. 10.1038/s41597-020-0448-0 32210236 PMC7093412

[98] Ponce-de-Leon M Del Valle J Fernandez JM Bernardo M Cirillo D Sanchez-Valle J. COVID-19 flow-maps an open geographic information system on COVID-19 and human mobility for Spain. Sci Data. (2021) 8:1–16. 10.1038/s41597-021-01093-5 34848723 PMC8633006

[99] Eubank S Guclu H Kumar VA Marathe MV Srinivasan A Toroczkai Z. Modelling disease outbreaks in realistic urban social networks. Nature. (2004) 429:180–4. 10.1038/nature02541 15141212

[100] Eubank S Kumar VA Marathe MV Srinivasan A Wang N. Structure of social contact networks and their impact on epidemics. DIMACS Ser Disc Math Theor Comput Sci. (2006) 70:181. 10.1090/dimacs/070/09

[101] Wang Z Meng C Yao M Claramunt C. Modelling the risk of imported COVID-19 infections at maritime ports based on the mobility of international-going ships. ISPRS Int J Geo-Inform. (2022) 11:60. 10.3390/ijgi11010060

[102] Almagor J Picascia S. Exploring the effectiveness of a COVID-19 contact tracing app using an agent-based model. Sci Rep. (2020) 10:1–11. 10.1038/s41598-020-79000-y 33335125 PMC7746740

[103] Natividade MS Bernardes K Pereira M Miranda SS Bertoldo J Teixeira MG. Social distancing and living conditions in the pandemic COVID-19 in Salvador-Bahia, Brazil. Ciência Saúde Coletiva. (2020) 25:3385–92. 10.1590/1413-81232020259.22142020 32876242

[104] Liu D Zheng X Zhang LJL. Simulation of spatiotemporal relationship between COVID-19 propagation and regional economic development in China. Land. (2021) 10:599. 10.3390/land10060599

[105] Diaz-Aviles E Stewart A Velasco E Denecke K Nejdl W. “Epidemic intelligence for the crowd, by the crowd,” in *Proceedings of the International AAAI Conference on Web and Social Media*. Dublin, Ireland (2012).

[106] Yang T Shen K He S Li E Sun P Chen P., CovidNet: to bring data transparency in the era of COVID-19. arXiv preprint arXiv:2005.10948 (2020). 10.48550/arXiv.2005.10948

[107] Zhou L Wu S Zhou M Li F. “School's out, but class' on”, the largest online education in the world today: taking China's practical exploration during the COVID-19 epidemic prevention and control as an example. Best Evid Chin Edu. (2020) 4:501–19. 10.15354/bece.20.ar023

[108] Li D Shao Z Yu W Zhu X Zhou S. Public epidemic prevention and control services based on big data of spatiotemporal location make cities more smart. Geomat Inform Sci Wuhan Univ. (2020) 45:475–87. 10.13203/j.whugis20200145

[109] Huang WB Ao S Han D Liu YM Liu S Huang YJ. Data-driven and machine-learning methods to project coronavirus disease 2019 pandemic trend in Eastern Mediterranean. Front Public Health. (2021) 9:9. 10.3389/fpubh.2021.602353 34055708 PMC8158576

[110] Yahya BM Yahya FS Thannoun RG. COVID-19 prediction analysis using artificial intelligence procedures and GIS spatial analyst: a case study for Iraq. Appl Geomat. (2021) 13:481–91. 10.1007/s12518-021-00365-4

[111] Evaluation IFHMA,. COVID-19 Projections. (2022). Available online at: https://covid19.healthdata.org/global?view=cumulative-deaths&tab=trend (accessed June 22, 2022).

[112] Kim J Kwan M-P. The impact of the COVID-19 pandemic on people's mobility: a longitudinal study of the US from March to September of 2020. J Transp Geogr. (2021) 93:103039. 10.1016/j.jtrangeo.2021.103039 PMC9759208 36569218

[113] Huang J Kwan M-P Kan Z Wong MS Kwok CYT Yu X. Investigating the relationship between the built environment and relative risk of COVID-19 in Hong Kong. ISPRS Int J Geo-Inform. (2020) 9:624. 10.3390/ijgi9110624

[114] Kan ZH Kwan MP Wong MS Huang JW Liu D. Identifying the space-time patterns of COVID-19 risk and their associations with different built environment features in Hong Kong. Sci Total Environ. (2021) 772:12. 10.1016/j.scitotenv.2021.145379 33578150 PMC7839428

[115] Ma J Zhu H Li P Liu C Li F Luo Z. Spatial patterns of the spread of COVID-19 in Singapore and the influencing factors. ISPRS Int J Geo-Inform. (2022) 11:152. 10.3390/ijgi11030152

[116] Kwok CYT Wong MS Chan KL Kwan M-P Nichol JE Liu CH. Spatial analysis of the impact of urban geometry and socio-demographic characteristics on COVID-19, a study in Hong Kong. Sci Total Environ. (2021) 764:144455. 10.1016/j.scitotenv.2020.144455 33418356 PMC7738937

[117] Guo Z Liu X Zhao P. A vector field approach to estimating environmental exposure using human activity data. ISPRS Int J Geo-Inform. (2022) 11:135. 10.3390/ijgi11020135

[118] Landi F Gremese E Bernabei R Fantoni M Gasbarrini A Settanni CR. Post-COVID-19 global health strategies: the need for an interdisciplinary approach. Aging Clin Exp Res. (2020) 32:1613–20. 10.1007/s40520-020-01616-x 32529595 PMC7287410

[119] Bontempi E Vergalli S Squazzoni F. Understanding COVID-19 diffusion requires an interdisciplinary, multi-dimensional approach. Environ Res. (2020) 188:109814. 10.1016/j.envres.2020.109814 32544726 PMC7289085

[120] Su Y Li Y Liu Y. Common demand vs. limited supply—how to serve the global fight against COVID-19 through proper supply of COVID-19 vaccines. Int J Environ Res. (2022) 19:1339. 10.3390/ijerph19031339 35162361 PMC8834692

[121] Telenti A Arvin A Corey L Corti D Diamond MS García-Sastre A. After the pandemic: perspectives on the future trajectory of COVID-19. Nature. (2021) 596:495–504. 10.1038/s41586-021-03792-w 34237771

[122] Lee J-G Kang M. Geospatial big data: challenges and opportunities. Big Data Res. (2015) 2:74–81. 10.1016/j.bdr.2015.01.003

[123] Shi P Dong Y Yan H Zhao C Li X Liu W. Impact of temperature on the dynamics of the COVID-19 outbreak in China. Sci Total Environ. (2020) 728:138890. 10.1016/j.scitotenv.2020.138890 32339844 PMC7177086

[124] Xie J Zhu Y. Association between ambient temperature and COVID-19 infection in 122 cities from China. Sci Total Environ. (2020) 724:138201. 10.1016/j.scitotenv.2020.138201 32408450 PMC7142675

[125] Chen MJ Chen YM Wilson JP Tan HY Chu TY. Using an eigenvector spatial filtering-based spatially varying coefficient model to analyze the spatial heterogeneity of COVID-19 and its influencing factors in mainland China. ISPRS Int J Geo-Inform. (2022) 11:21. 10.3390/ijgi11010067

[126] Flaxman S Mishra S Gandy A Unwin HJT Mellan TA Coupland H. Estimating the effects of non-pharmaceutical interventions on COVID-19 in Europe. Nature. (2020) 584:257–61. 10.1038/s41586-020-2405-7 32512579

[127] Hsiang S Allen D Annan-Phan S Bell K Bolliger I Chong T. The effect of large-scale anti-contagion policies on the COVID-19 pandemic. Nature. (2020) 584:262–7. 10.1038/s41586-020-2404-8 32512578

[128] Chen X Zhang A Wang H Gallaher A Zhu X. Compliance and containment in social distancing: mathematical modeling of COVID-19 across townships. Int J Geogr Inform Sci. (2021) 2021:1–20. 10.1101/2020.06.01.20119073

[129] Spotswood EN Benjamin M Stoneburner L Wheeler MM Beller EE Balk D. Nature inequity and higher COVID-19 case rates in less-green neighbourhoods in the United States. Nat Sustain. (2021) 2021:1–7. 10.1038/s41893-021-00781-9

